# Maternal SARS-CoV-2 infection elicits sexually dimorphic placental immune responses

**DOI:** 10.1126/scitranslmed.abi7428

**Published:** 2021-10-27

**Authors:** Evan A. Bordt, Lydia L. Shook, Caroline Atyeo, Krista M. Pullen, Rose M. De Guzman, Marie-Charlotte Meinsohn, Maeva Chauvin, Stephanie Fischinger, Laura J. Yockey, Kaitlyn James, Rosiane Lima, Lael M. Yonker, Alessio Fasano, Sara Brigida, Lisa M. Bebell, Drucilla J. Roberts, David Pépin, Jun R. Huh, Staci D. Bilbo, Jonathan Z. Li, Anjali Kaimal, Danny J. Schust, Kathryn J. Gray, Douglas Lauffenburger, Galit Alter, Andrea G. Edlow

**Affiliations:** 1 Department of Pediatrics, Lurie Center for Autism, Massachusetts General Hospital, Harvard Medical School, Boston, MA 02129, USA.; 2 Department of Obstetrics and Gynecology, Massachusetts General Hospital, Harvard Medical School, Boston, MA 02114, USA.; 3 Vincent Center for Reproductive Biology, Massachusetts General Hospital, Boston, MA 02114, USA.; 4 Ragon Institute of MGH, MIT, and Harvard, Cambridge, MA 02139, USA.; 5 PhD Program in Virology, Division of Medical Sciences, Harvard University, Boston, MA 02115, USA.; 6 Department of Biological Engineering, Massachusetts Institute of Technology, Cambridge, MA 02142, USA.; 7 Pediatric Surgical Research Laboratories, Department of Surgery, Massachusetts General Hospital, Harvard Medical School, Boston, MA 02114, USA.; 8 Department of Medicine, Massachusetts General Hospital, Harvard Medical School, Boston, MA 02114, USA.; 9 Mucosal Immunology and Biology Research Center, Department of Pediatrics, Massachusetts General Hospital, Boston, MA 02129, USA.; 10 European Biomedical Research Institute of Salerno (EBRIS), Salerno, Italy.; 11 Department of Pathology, Massachusetts General Hospital, Harvard Medical School, Boston, MA 02114, USA.; 12 Department of Immunology, Blavatnik Institute, Harvard Medical School, Boston, MA 02115, USA.; 13 Evergrande Center for Immunologic Diseases, Harvard Medical School and Brigham and Women’s Hospital, Boston, MA 02115, USA.; 14 Department of Psychology and Neuroscience, Duke University, Durham, NC 27708, USA.; 15 Department of Medicine, Brigham and Women’s Hospital, Boston, MA 02115, USA.; 16 Department of Obstetrics, Gynecology, and Women’s Health, University of Missouri, Columbia, MO 65201, USA.; 17 Department of Obstetrics and Gynecology, Brigham and Women’s Hospital, Harvard Medical School, Boston, MA 02115, USA.

## Abstract

There is a persistent bias toward higher prevalence and increased severity of coronavirus disease 2019 (COVID-19) in males. Underlying mechanisms accounting for this sex difference remain incompletely understood. Interferon responses have been implicated as a modulator of COVID-19 disease in adults and play a key role in the placental antiviral response. Moreover, the interferon response has been shown to alter Fc receptor expression and therefore may affect placental antibody transfer. Here, we examined the intersection of maternal-fetal antibody transfer, viral-induced placental interferon responses, and fetal sex in pregnant women infected with severe acute respiratory syndrome coronavirus 2 (SARS-CoV-2). Placental Fc receptor abundance, interferon-stimulated gene (ISG) expression, and SARS-CoV-2 antibody transfer were interrogated in 68 human pregnancies. Sexually dimorphic expression of placental Fc receptors, ISGs and proteins, and interleukin-10 was observed after maternal SARS-CoV-2 infection, with up-regulation of these features in placental tissue of pregnant individuals with male fetuses. Reduced maternal SARS-CoV-2–specific antibody titers and impaired placental antibody transfer were also observed in pregnancies with a male fetus. These results demonstrate fetal sex-specific maternal and placental adaptive and innate immune responses to SARS-CoV-2.

## INTRODUCTION

Mortality and morbidity risk during the perinatal period and infancy is higher in males than in females (1–4). The underlying susceptibility of males may relate to evolutionary differences that occur throughout pregnancy and in the perinatal period, but the precise mechanistic differences that lead to this differential female survival benefit are not completely understood. Consistent with perinatal male vulnerability in general, male infants and children fare worse in the setting of severe acute respiratory syndrome coronavirus 2 (SARS-CoV-2) infection, with higher rates of severe disease in infants and of SARS-CoV-2–associated multisystem inflammatory syndrome (MIS-C) in male children (5–10). The biological basis for the observed relative vulnerability of the male immune system to SARS-CoV-2 in pediatric populations is likely multifactorial (11, 12). Emerging data point to a retrograde impact of infant sex on maternal immunity (13, 14), with specific differences in innate immune signaling across fetal sex, which may contribute to a differential dialogue between female and male fetuses and their mothers. This differential dialogue may critically affect immunity across the dyad, pointing to one potential explanation for sex differences in perinatal vulnerability to infectious disease: differential transplacental antibody transfer from the mother may provide female and male infants with different degrees of immunity.

Newborn antiviral immunity relies heavily on the placental transfer of maternal immunoglobulin G (IgG) to the fetal circulation (15–17). Public health strategies to protect newborns from potentially devastating respiratory infections such as pertussis and influenza capitalize on the ability of the placenta to transfer vaccine-induced maternal IgG to the fetal circulation (18, 19). Although the neonatal Fc receptor (FcRn) was classically identified as the primary receptor responsible for transferring maternal IgG to fetal circulation (20–22), recent findings have also demonstrated critical roles for the Fc-γ receptors (FCγRs) I, II and III in facilitating maternal IgG transfer (15, 16, 23–25). FCγRI expression is regulated by type I and II interferons (IFNs), and emerging data have clearly demonstrated perturbed placental transfer in the setting of other coinfections, including HIV (26) and malaria (27). Whether differences in inflammatory responses to SARS-CoV-2 infection could influence placental antibody transfer is unknown. In addition, little is known regarding sex differences in neonatal immune profiles and in maternal-fetal antibody transfer. Recent work has demonstrated reduced transplacental transfer of SARS-CoV-2–specific antibodies relative to influenza and pertussis antibodies (28, 29) and associated alterations in expression and localization of specific Fc receptors in the placenta (29), but sex differences in neonatal antibody-mediated immunity to SARS-CoV-2 and in placental receptors involved in antibody transfer have not yet been characterized.

Type I, type II, and type III IFNs are induced after innate recognition of viruses (30). Upon binding to their receptors, they induce expression of downstream effectors, IFN-stimulated genes (ISGs), which inhibit viral infection by a number of different mechanisms (31). However, viruses have evolved to evade these IFN responses, and IFN responses can also be drivers of inflammatory pathology. Type I IFN signaling correlates strongly with pathogenicity and fatality in both SARS-CoV-1 and Middle East respiratory syndrome coronavirus (MERS-CoV) infections (32–34). Dysregulated type I IFN signaling is also associated with severe disease and drives pathogenicity in SARS-CoV-2 infection in both humans and murine models (33, 35–41). Sex differences in adult peripheral blood and pulmonary IFN signaling have been observed in both SARS-CoV-1 and SARS-CoV-2 infection (42–44), but there is a dearth of information about sex differences in fetal and pediatric populations. Type I and type III IFN responses at the maternal-fetal interface play a crucial role in limiting viral infection but may also be drivers of abnormal development (45–47). Less is known about the role of type II IFN signaling (initiated by IFN-γ) in the placenta and in SARS-CoV-2 infection (48–53). Sex differences have been noted in the placental immune response to bacterial infection (54, 55) and to other prenatal alterations such as maternal stress and maternal high-fat diet (12, 56, 57). Placental expression of ISGs in maternal SARS-CoV-2 infection, the role of sex differences, and potential impact on placental function have not yet been examined.

Given known sex differences in immune responses to SARS-CoV-2 infection (42), the observed sex differences in disease prevalence and severity in the pediatric population including infants (6–9), and the placenta’s critical role as an immune organ mediating antiviral responses and antibody transfer at the maternal-fetal interface (47), we sought to examine sex differences in the placental immune response to SARS-CoV-2 and how this may affect placental expression of receptors associated with antibody transfer. In a cohort of 38 participants infected with SARS-CoV-2 during pregnancy (19 male and 19 female fetuses) and a comparator group of 30 contemporaneously enrolled pregnant women testing negative for SARS-CoV-2 (15 male and 15 female fetuses), notable differences were noted in placental transfer of antibodies to male and female infants, as well as sexually dimorphic placental Fc receptor expression and ISG and IFN-stimulated protein expression in the setting of maternal SARS-CoV-2 infection. These data point to unexpected sex-driven bilateral communication across the maternal-fetal interface, associated with sex differences in SARS-CoV-2–specific antibody transfer that may provide insight into sex-biased differences in susceptibility to infectious diseases in male infants.

## RESULTS

### Demographic and clinical characteristics of study participants

Maternal demographic and clinical characteristics of study participants for placental analyses grouped by offspring sex are depicted in Table 1, and those providing matched maternal and umbilical cord blood are depicted in table S1. Of the 68 participants, 34 were pregnant with females and 34 with males. There were no differences between groups with respect to maternal age, parity, obesity, diabetes, hypertension, or gestational age at delivery, among other characteristics examined. Women with SARS-CoV-2 infection during pregnancy were more likely to be Hispanic compared to negative controls, concordant with our prior report of ethnic disparities in coronavirus disease 2019 (COVID-19) vulnerability in our hospital catchment area (58). Of the 38 women with SARS-CoV-2 infection during pregnancy, there were no differences between male and female fetuses with respect to gestational age at diagnosis of SARS-CoV-2 infection, days from positive SARS-CoV-2 test to delivery, or severity of COVID-19 illness. Although both male and female fetuses of mothers with SARS-CoV-2 had lower birthweights than their sex-matched control counterparts, clinically, this difference was not meaningful as all neonatal birthweights were in the normal range. No neonates born to mothers with SARS-CoV-2 infection during pregnancy were infected with SARS-CoV-2.

### Sex differences were observed in placental transfer of SARS-CoV-2–specific antibodies and antibody function

**Fig. 1.**
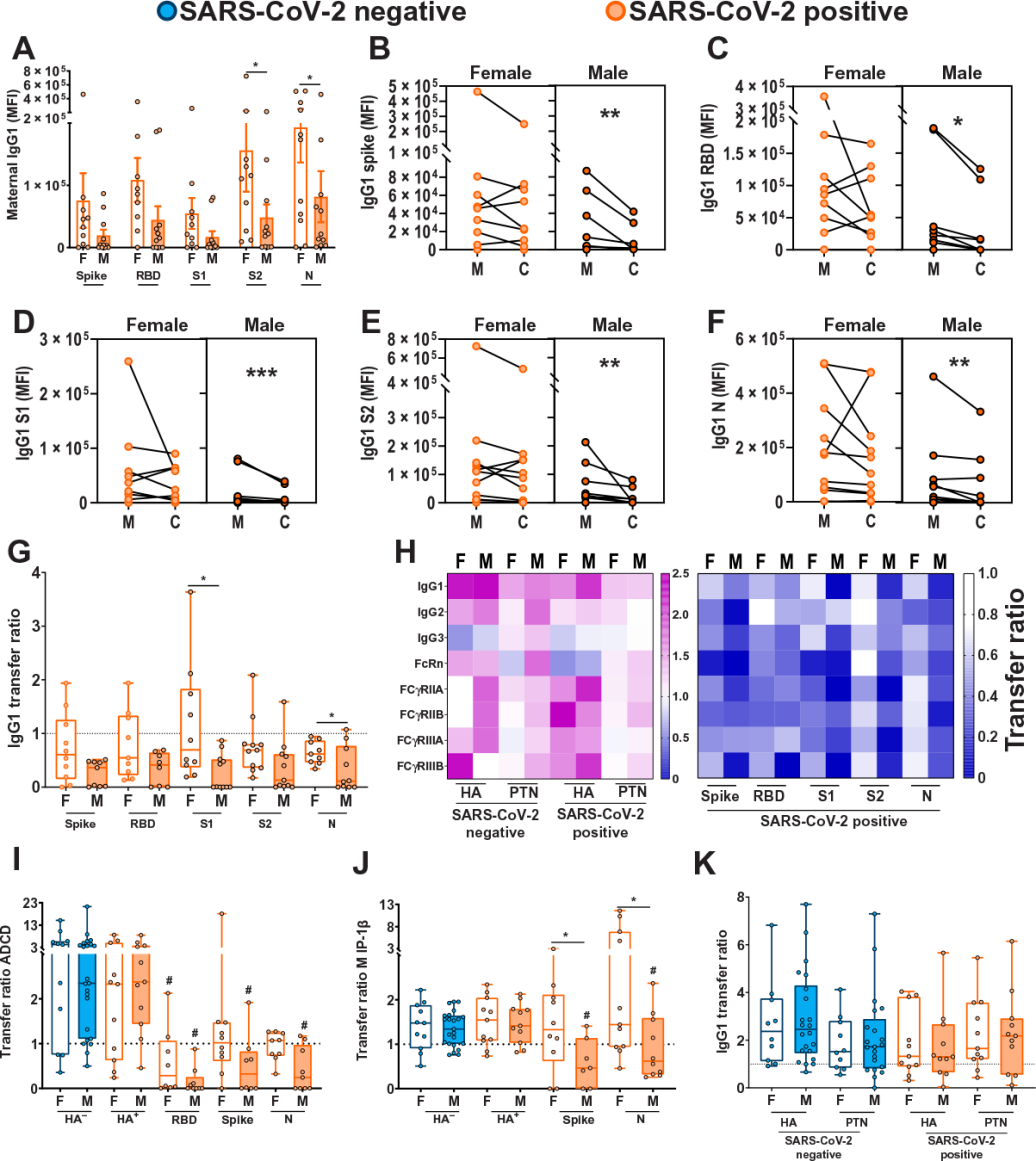
SARS-CoV-2–positive mothers with male fetuses demonstrate reduced maternal titers and placental transfer of SARS-CoV-2–specific antibodies compared to those with female fetuses. (**A**) Bar graphs depict spike protein–, RBD-, S1-, S2-, and N protein–specific maternal IgG1 titers (*n* = 11 per group). F, female fetus; M, male fetus. Differences across groups were assessed by two-way ANOVA followed by Bonferroni’s post hoc analyses. There was a main effect of fetal sex on maternal IgG1 titers. MFI, median fluorescence intensity. *P < 0.05. (**B** to **F**) Dot plots showing relative spike protein– (B), RBD- (C), S1- (D), S2- (E), and N protein–specific (F) maternal blood (M) and cord blood (C) titers of IgG1 (*n* = 11 per group). Female neonates of SARS-CoV-2–positive mothers are shown in light orange. Males born to SARS-CoV-2–positive mothers are shown in dark orange. All values reflect phosphate-buffered saline (PBS) background correction. Group differences were assessed by Wilcoxon matched-pairs signed rank test. *P < 0.05, **P < 0.01, and ***P < 0.001. (**G**) Box and whisker plots show the placental transfer ratios (cord:maternal ratios) for IgG1 against the SARS-CoV-2 antigens spike protein, RBD, S1, S2, and N. SARS-CoV-2–positive maternal status is shown in orange [female (F), open bars; male (M), shaded bars]. All values are PBS background corrected (*n* = 11 per group). Differences across groups were assessed by Kruskal-Wallis test followed by Dunn’s post hoc analyses. Dotted line denotes a transfer ratio of 1 or 100%. *P < 0.05. (**H**) Heatmap depicting the median PBS background–corrected cord:maternal transfer ratio of HA, PTN, spike protein, RBD, S1, S2, and N protein across all antibody subclasses and Fc receptor binding profiles in both SARS-CoV-2–negative and SARS-CoV-2–positive maternal:neonate dyads. (**I** and **J**) Box and whisker plots depicting transfer ratios (cord:maternal) for HA-, RBD-, spike protein–, or N protein–specific antibodies (*n* = 11 to 23 per group) mediating antibody-dependent complement deposition [ADCD (I)] and macrophage inflammatory protein-1β (MIP-1β) expression (J). SARS-CoV-2–negative maternal status is shown in blue (female, open bars; male, shaded bars), and SARS-CoV-2–positive maternal status is shown in orange (female, open bars; male, shaded bars). HA^−^ indicates presence of antibodies specific to HA antigen in SARS-CoV-2–negative pregnancies, HA^+^ indicates presence of antibodies specific to HA antigen in SARS-CoV-2–positive pregnancies. Differences across groups were assessed by Kruskal-Wallis test followed by Dunn’s post hoc analyses. For each SARS-CoV-2 antigen, post hoc analyses were performed against the HA-specific activity using the matched SARS-CoV-2–positive dyad. #*P* < 0.01 compared to HA transfer ratio for SARS-CoV-2–positive samples of that sex, and *P < 0.01 compared to indicated group. (**K**) The box and whisker plots show the transfer ratios (cord:maternal ratio) for IgG1 against HA and PTN in maternal:neonate dyads from either SARS-CoV-2–negative or SARS-CoV-2–positive pregnancies (*n* = 11 to 23 per group). SARS-CoV-2 negative are shown in blue (female, open bars; male, shaded bars), and SARS-CoV-2 positive are shown in orange (female, open bars; male, shaded bars). Differences across groups were assessed by Kruskal-Wallis test followed by Dunn’s post hoc analyses. In (I) to (K), dotted line denotes transfer ratio of 1 or 100%. *P < 0.05. For box and whisker plots in (A), (G), and (I) to (K), the box extends from the 25th to 75th percentile, the whiskers depict minimum and maximum, and horizontal line depicts the median.

Reduced transplacental antibody transfer has been observed in the context of maternal infections such as HIV (26) and malaria (27), and our group and others have previously described deficits in transplacental antibody transfer of SARS-CoV-2–specific antibodies (28, 29, 59, 60). Given the known vulnerability of male infants to more severe respiratory disease (11), we sought to determine whether the sex of the fetus would affect transplacental transfer of maternal SARS-CoV-2–specific antibodies. We comprehensively profiled SARS-CoV-2 antibodies targeting the spike protein (S), the receptor binding domain (RBD), the spike S1 subunit (S1), the spike S2 subunit (S2), and the nucleocapsid protein (N) including titer, function, and placental receptor interactions in maternal-cord plasma pairs of SARS-CoV-2–exposed and SARS-CoV-2–unexposed (SARS-CoV-2–negative) pregnancies, using a previously described systems serology approach (29, 61). Maternal IgG titers against SARS-CoV-2–specific antigens, an important driver of maternal-fetal immune transfer (62, 63), were significantly lower in mothers carrying a male fetus (Fig. 1A and fig. S1; *P* < 0.05). Rather than the expected transplacental transfer observed for other pathogens [cord-to-maternal ratio of 1.1 to 1.5 or greater (24, 64, 65)], IgG titers against all examined SARS-CoV-2 antigens were significantly reduced in cord relative to maternal plasma across IgG subtypes in pregnancies with a male fetus compared with those with a female fetus (Fig. 1, B to F, and fig. S2; *P* < 0.05, *P* < 0.01, or *P* < 0.001). In contrast, significant reduction in cord versus maternal titers was only observed in the case of N protein–specific IgG2 in pregnancies with a female fetus (fig. S2, *P* < 0.05). There were significantly reduced cord:maternal transfer ratios for SARS-CoV-2–specific antibodies in male versus female fetuses (Fig. 1G; *P* < 0.05) and reduced transfer of SARS-CoV-2–specific antibodies capable of binding to FcRn, FCγRIIA/B, and FCγRIIIA/B predominantly in males (Fig. 1H and fig. S2). Although transfer ratios for SARS-CoV-2–specific antibodies were higher for female fetuses than those for males, they were still lower than the expected 1.1 to 1.5 ratios often observed for other pathogens (29, 64, 65). SARS-CoV-2–specific functional antibodies were also transferred less efficiently, with a marked male-specific decrease in antibody-dependent complement deposition (ADCD)–inducing antibodies and antibodies that induce natural killer (NK) cell chemokine secretion [macrophage inflammatory protein-1β (MIP-1β); Fig. 1, I and J].

In contrast to SARS-CoV-2–specific antibodies, efficient transfer of pertussis antigen pertactin (PTN) and the influenza hemagglutinin (HA) glycoprotein-specific titers (Fig. 1K and fig. S3) and functions (Fig. 1, I and J) was observed across pregnancies with both female and male fetuses. Timing from maternal vaccination to sample collection for titer determination is depicted in table S2. In addition to influenza and pertussis antibodies, which reflect maternal vaccination during pregnancy, we assessed the transfer of antibodies against endemic, chronic, and other childhood vaccinatable pathogens including measles, mumps, rubella, varicella zoster virus (VZV), common coronaviruses NL63 and OC43, cytomegalovirus (CMV), and Epstein-Barr virus (EBV) and found no sex differences in maternal IgG1 titers for these pathogens (fig. S4). We did, however, identify increased transplacental transfer of anti-measles, anti-mumps, anti-VZV IgG1, and anti-NL63 IgG1 in females but not males exposed to maternal SARS-CoV-2, relative to sex-matched controls (fig. S4A). Although significantly lower maternal titers of SARS-CoV-2–specific IgG1 were observed in the setting of a male fetus (Fig. 1A; *P* < 0.05), this effect was specific to anti–SARS-CoV-2 antibodies, with no impact of fetal sex on maternal anti-HA, PTN, tetanus, VZV, CMV, measles, mumps, rubella, NL63, OC43, or EBV titers (fig. S4, B and C). Similar results were observed for maternal IgG2, IgG3, and IgM (fig. S5). Overall, these results suggest a reduced maternal SARS-CoV-2–specific humoral immune response in pregnancies with male fetuses, consistent with prior studies demonstrating suppressed maternal proinflammatory responses in mothers with a male fetus relative to a female fetus (13, 14) and the known direct correlation between proinflammatory response and increased antibody production in COVID-19 infection (66, 67).

### Sexually dimorphic placental Fc receptor expression was observed in response to maternal SARS-CoV-2 infection

**Fig. 2.**
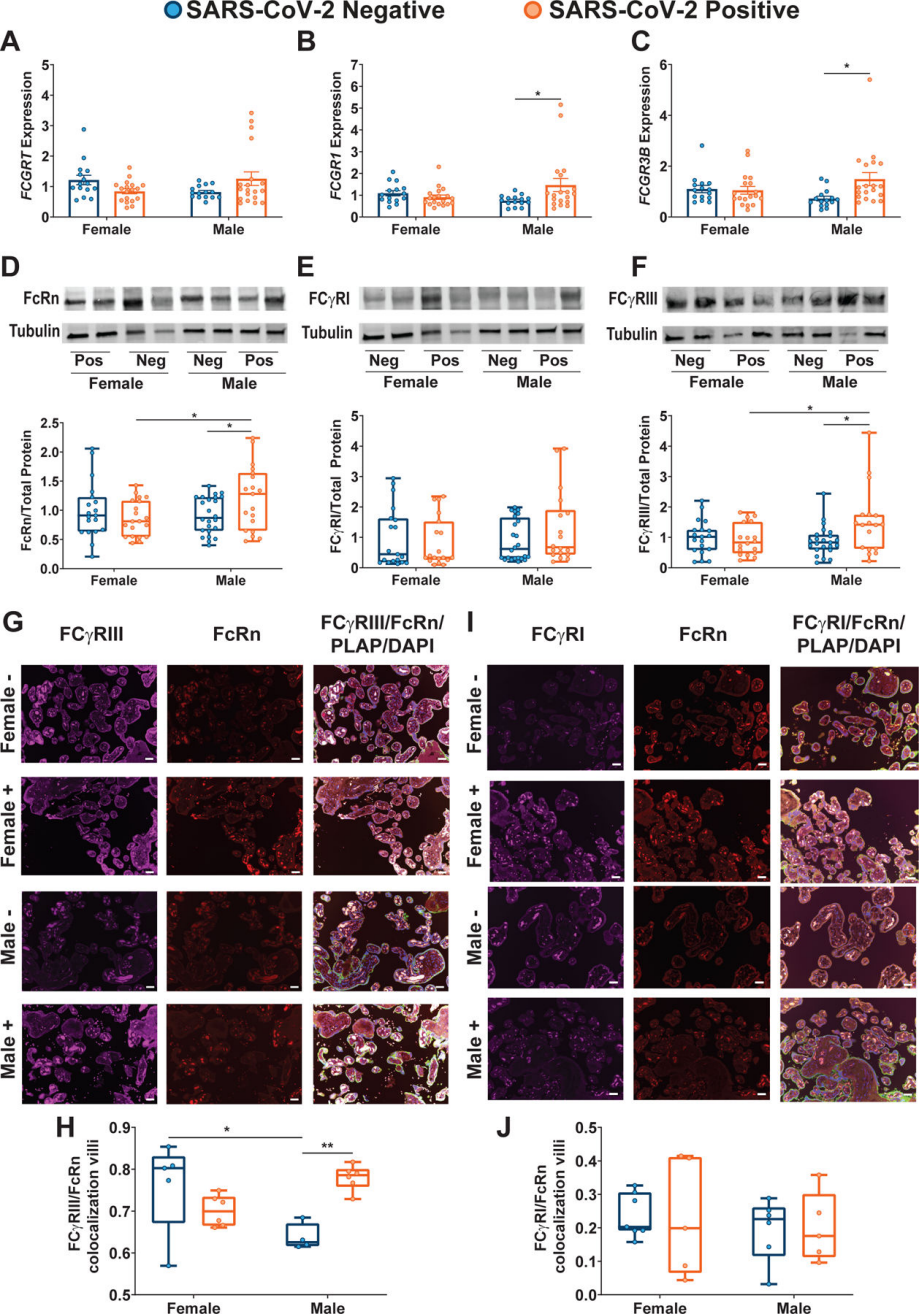
Sexually dimorphic regulation of placental Fc receptor gene and protein expression is observed in the setting of maternal SARS-CoV-2 infection. (**A** to **C**) RT-qPCR analyses of male or female placental expression of *FCGRT* (A), *FCGR1* (B), and *FCGR3B* (C) in placental biopsies from SARS-CoV-2–negative (blue) or SARS-CoV-2–positive (orange) pregnancies (*n* = 15 to 18 per group). Expression shown is relative to reference genes *YWHAZ* and *TOP1*. Bar plots in (A) to (C) indicate means ± SEM. (**D** to **F**) Representative immunoblots and quantification of fetal female or fetal male expression of FcRn (D), FCγRI (E), and FCγRIII (F) in placental biopsies from SARS-CoV-2–negative (blue) or SARS-CoV-2–positive (orange) pregnancies (*n* = 19 per group). Neg and Pos on Western blot designates SARS-CoV-2–negative and SARS-CoV-2–positive pregnancies, respectively. For box and whisker plots in (D) to (F), the box extends from the 25th to 75th percentile, the whiskers depict minimum and maximum, and horizontal line depicts the median. (**G**) Placental tissue sections from SARS-CoV-2–positive and SARS-CoV-2–negative mothers were stained for FCγRIII (purple), FcRn (red), and placental alkaline phosphatase (PLAP; green), a trophoblast marker, and 4′,6-diamidino-2-phenylindole (DAPI, blue). (**H**) Box and whisker plots showing FCγRIII/FcRn colocalization in placental villi (*n* = 4 to 6 per group). (**I**) Placental tissue sections from SARS-CoV-2–positive and SARS-CoV-2–negative mothers were stained for FCγRI (purple), FcRn (red), and PLAP (green), a trophoblast marker, and DAPI (blue). (**J**) Box and whisker plots showing FCγRI/FcRn colocalization in placental villi (*n* = 5 to 7 per group). Scale bars, 100 μm (G and I). For box and whisker plots in (H) and (J), the box extends from the 25th to 75th percentile, the whiskers depict minimum and maximum, and horizontal line depicts the median. Differences across groups were assessed by two-way ANOVA followed by Bonferroni’s post hoc analyses. *P < 0.05 and **P < 0.01.

The transfer of maternal antibodies across the placenta is mediated by Fc receptors (15, 16). FcRn was considered the classical receptor mediating transplacental transfer of IgG (20–22), although other Fc receptors (FcγRI, FcγRII, and FcγRIII) are increasingly recognized as being expressed in the placenta and likely to play a role in transfer (15, 16, 23–25). We observed sexually dimorphic expression of *FCGRT*, *FCGR1*, and *FCGR3A/B* genes in the setting of maternal SARS-CoV-2 infection (Fig. 2, A to C, fig. S6, A to C, and table S3), driven by increased expression in male SARS-CoV-2–exposed placentas. There were no fetal sex– or maternal SARS-CoV-2 infection–mediated differences in placental expression of *FCGR2A/B* (fig. S6, B and C, and table S3). These findings point to sexual dimorphism in Fc receptor expression with up-regulation in male placentas. To distinguish whether increased transcript expression of Fc receptors was compensatory in the setting of diminished protein expression or whether protein expression mirrored gene expression, we performed immunoblot analyses of FcRn, FCγRIII, FCγRII, and FCγRI. Protein expression was consistent with gene expression for FcRn, FCγRII, and FCγRIII (Fig. 2, D to F, fig. S6D, and table S3). There was no impact of maternal SARS-CoV-2 infection or fetal sex on protein expression of FCγRI (Fig. 2E and table S3). In addition to Fc receptor quantity, it has been demonstrated that colocalization of other Fcγ receptors with FcRn typically augments efficiency of placental antibody transfer (16, 29). Immunohistochemical analyses of placental villi revealed sexual dimorphism in placental Fc receptor colocalization, with a significant increase in colocalization of FCγRIII and FcRn in male placentas only (Fig. 2, G and H, and table S3; *P* < 0.01). No fetal sex or maternal SARS-CoV-2 exposure differences in colocalization with FcRn were observed for FCγRI (Fig. 2, I and J, and table S3) or FCγRII (fig. S6, E to G, and table S3). The gene and protein expression results suggest that maternal SARS-CoV-2 infection has a sexually dimorphic impact on placental Fcγ receptor expression, driven by an increase in overall expression and increased FCγRIII/FcRn colocalization in male placentas. Increased placental Fc receptor expression was not sufficient to restore normal placental transfer of humoral immunity to male fetuses.

### Spike protein–specific Fc-glycan profiles coupled with placental Fc receptor expression patterns drive reduced placental antibody transfer in male pregnancies

**Fig. 3.**
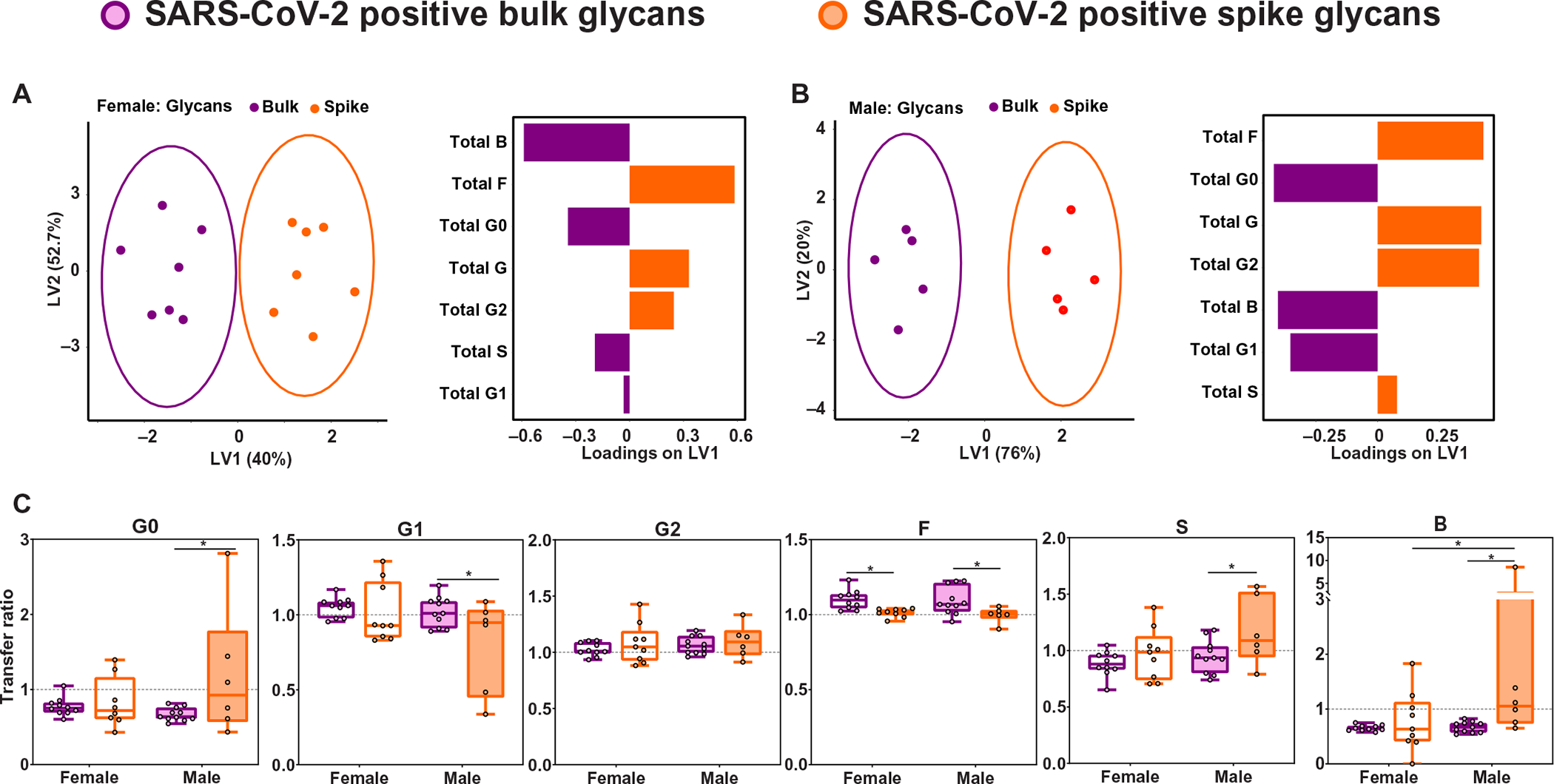
Sex differences in placental transfer are driven by differences in bulk versus spike protein–specific Fc-glycan transfer. (**A** and **B**) A multilevel orthogonal partial least squares discriminant analysis (O-PLSDA) was built using bulk (purple) and spike protein–specific (orange) antibody glycan data from SARS-CoV-2–positive mothers pregnant with a female fetus (A) or male fetus (B) for which matched data were available. Each maternal sample is depicted as a dot on the scores plot (left). The glycan feature loadings on the first latent variable (LV1) are depicted by the bar graph (right). The model performance was assessed using leave-one-out cross-validation, with the average accuracy scores reported to be 98 and 100% predictive accuracy for the female and male O-PLSDA models, respectively. (**C**) Box and whisker plots showing the transfer ratios (cord:maternal ratios) for bulk and spike protein–specific Fc-glycans. Glycoforms depicted are agalactosylated (G0), monogalactosylated (G1), digalactyosylated (G2), fucosylated (F), sialylated (S), and bisected *n*-acetyl-glucosamine (GlcNAc, B). Bulk glycans are shown in purple, and spike protein–specific glycans are shown in orange (*n* = 8 to 11 per group). Pregnancies with female neonates are shown as open bars, whereas pregnancies with male neonates are shown as shaded bars. For box and whisker plots in (C), box extends from the 25th to 75th percentile, the whiskers depict minimum and maximum, and horizontal line depicts the median. Differences across groups were assessed by two-way ANOVA followed by FDR multiple comparisons correction post hoc analyses. *P < 0.05.

Given that antibody glycosylation has been demonstrated to be a key driver of reduced SARS-CoV-2–specific antibody transfer in maternal-cord dyads (68), we next profiled glycosylation of bulk and spike protein–specific antibodies in SARS-CoV-2–positive pregnancies in the context of a male versus a female fetus. We identified significant differences between bulk and spike protein–specific glycosylation profiles, more pronounced in the context of a male fetus (Fig. 3, A and B; *P* < 0.05). Spike protein–specific Fc-glycan profiles in SARS-CoV-2–positive mothers were marked by enhanced fucosylation (F) and digalactosylation (G2), and reduced bisecting-*n*-acetyl-glucosamine (b-GlcNAc, B) and agalactosylation (G0) on spike protein–specific antibodies in pregnancies with both male and female fetuses (Fig. 3, A and B, and fig. S7). Given that fucosylated antibodies are not transferred efficiently through FCγRIII (69–71), the increased expression of FCγRIII and increased colocalization of FCγRIII and FcRn in placentas of SARS-CoV-2–positive male pregnancies may have contributed to impaired placental transfer of SARS-CoV-2–specific antibodies. Because of the attenuated transfer of fucosylated antibodies resulting from up-regulation of FCγRIII, males preferentially transferred afucosylated antibodies (such as G0 and B), which were relatively scarce among the spike protein–specific antibodies. In contrast, pregnancies with a female fetus had relatively higher maternal titers of SARS-CoV-2–specific antibodies and decreased FCγRIII placental expression, allowing for more efficient transplacental antibody transfer of the available Fc-glycan profile on spike protein–specific antibodies. Bulk antibodies (including HA, PTN, MMR, VZV, common coronaviruses, CMV, and EBV) had a different glycan profile from spike protein–specific antibodies, with bulk antibodies overall enriched for G0 and B (Fig. 3, A and B). Sex-specific differences were noted in the glycosylation profile of antibodies transferred from mother to neonate (Fig. 3C). Pregnancies with a male fetus demonstrated significantly increased transfer of agalactosylated (G0), sialyated (S), and bisected GlcNAc (B) spike protein–specific antibodies relative to bulk (Fig. 3C; *P* < 0.05). Although both male and female spike protein–specific transfer of fucosylated (F) antibodies was decreased relative to bulk transfer, only males had impaired transfer of galactosylated (G1) antibodies (Fig. 3C). A possible etiology for the fundamental Fc-glycan differences between spike protein–specific and bulk antibodies within SARS-CoV-2–exposed dyads is the production of SARS-CoV-2–specific antibodies during a de novo infection in pregnancy. Inflammation has been demonstrated to alter the glycosylation profile of antibodies produced during that episode (72) and may be a driver of the differences noted in glycosylation profile of bulk compared with spike protein–specific antibodies.

### Sexually dimorphic placental expression of ISGs were observed in response to maternal SARS-CoV-2 infection

**Fig. 4.**
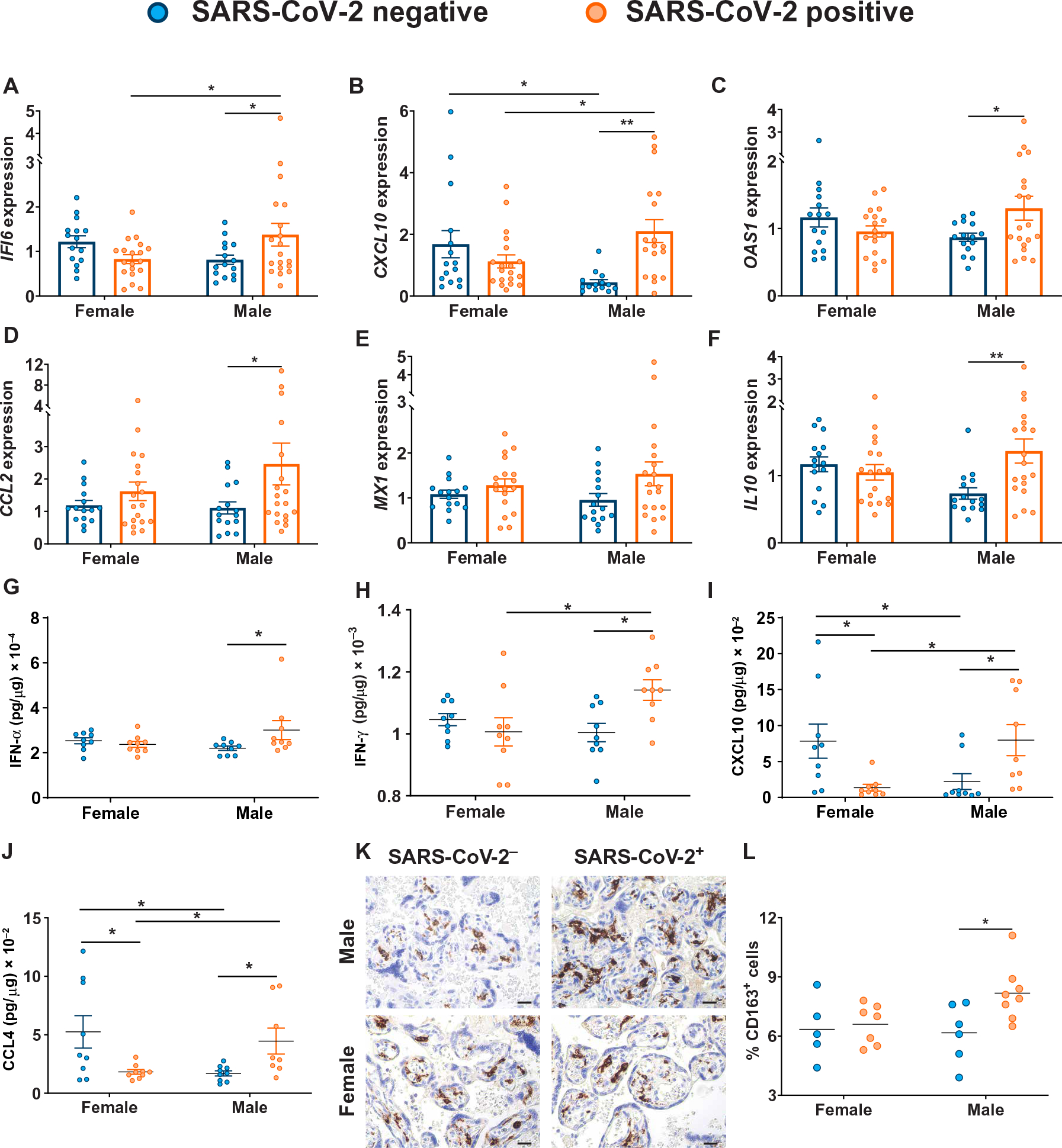
Male-specific up-regulation of ISGs in placentas exposed to maternal SARS-CoV-2 infection. (**A** to **F**) RT-qPCR analyses of male or female placental expression of *IFI6* (A), *CXCL10* (B), *OAS1* (C), *CCL2* (D), *MX1* (E), and *IL10* (F) in placental biopsies from SARS-CoV-2–negative (blue) or SARS-CoV-2–positive (orange) pregnancies (*n* = 15 to 18 per group). Expression shown is relative to reference genes *YWHAZ* and *TOP1.* Bar plots in (A) to (F) indicate means ± SEM. (**G** to **J**) Placental homogenate protein concentrations of IFN-α (G), IFN-γ (H), CXCL10 (I), and CCL4 (J) were quantified by Luminex multiplex assay (*n* = 9 per group). Data are presented as means ± SEM. (**K** and **L**) Representative immunohistochemistry images (K) and quantification (L) of CD163-positive cells (brown) in placental sections from SARS-CoV-2–negative (blue) or SARS-CoV-2–positive (orange) pregnancies are shown (*n* = 5 to 8 per group). Scale bars, 100 μm (K). Horizontal bars in (L) indicate mean. Differences across groups were assessed by two-way ANOVA followed by Bonferroni’s post hoc analyses. *P < 0.05 and **P < 0.01

Given the key role ISGs are known to play in the placental antiviral response (46, 47, 73) and placental barrier function (74), and previous reports of sex differences in expression of IFN-stimulated proteins in the plasma of patients with COVID-19 (42), we examined whether maternal SARS-CoV-2 infection was associated with sex-specific alterations in placental type I, II, and III IFN pathways (fig. S8A). There was a sexually dimorphic expression pattern of the classical ISGs *IFI6*, *CXCL10*, and *OAS1* driven by increased expression in male SARS-CoV-2–exposed placentas compared with SARS-CoV-2–negative controls (Fig. 4, A to C, and table S4). We next examined expression of *CCL2/MCP-1*, a type I IFN–stimulated cytokine implicated in monocyte chemotaxis (75) and up-regulated in lung samples from patients with COVID-19 (39, 40), as well as *MX1*, an antiviral response gene induced by type I/III IFNs and up-regulated during SARS-CoV-2 infection (76). Maternal SARS-CoV-2 exposure was associated with increased expression of *CCL2* and *MX1* in the placenta, with these differences primarily driven by increases in male placentas (Fig. 4, D and E, and table S4). Although maternal SARS-CoV-2 infection did not affect expression of placental *TNF*, *IL6*, or *CCL7* compared with expression in placentas from women negative for SARS-CoV-2 (fig. S8, B to D, and table S4), there was a sexually dimorphic effect of maternal SARS-CoV-2 infection on the expression of anti-inflammatory factor *IL10* (77), driven by significantly increased expression in SARS-CoV-2–exposed male placentas (Fig. 4F and table S4; *P* < 0.01). No changes in expression of the reference genes *YWHAZ* or *TOP1* were observed across fetal sex or maternal SARS-CoV-2 status groups (fig. S9 and table S4).

To confirm that the sexually dimorphic ISG response was translated at the protein level, we next assessed protein expression within the type I, II, and III IFN–stimulated pathways using a multiplex immunoassay. There was sexually dimorphic expression of placental IFN-α and IFN-γ, driven by increased IFN expression in male SARS-CoV-2–exposed placentas compared with SARS-CoV-2–negative controls (Fig. 4, G to H). Consistent with our gene expression profiling, we confirmed sexually dimorphic response of IFN-stimulated proteins CXCL10, CCL3, and CCL4 to maternal SARS-CoV-2 infection, although protein expression of CCL2 was not different, in contrast to what was observed at the gene level (Fig. 4, I and J, fig. S10, and table S4). Expression of some ISGs and proteins, in particular CXCL10 and CCL4, was also significantly lower in male compared with female control placentas in the absence of SARS-CoV-2 infection (Fig. 4, B, I, and J; *P* < 0.05). Protein analyses confirmed the lack of sex-specific alterations in placental expression of tumor necrosis factor-α (TNF-α), interleukin-6 (IL-6), IL-12p70, IL-13, IL-17A, IL-8, IL-1α, IL-1β, IL-4, CD62E, or CD62P in the setting of maternal SARS-CoV-2 infection (fig. S10 and table S4).

We also observed a male-specific increase in density of CD163^+^ Hofbauer cells, placental resident fetal macrophages (78), in response to maternal SARS-CoV-2 exposure (Fig. 4, K and L, and table S4). Although placental Hofbauer cell hyperplasia has been described in maternal SARS-CoV-2 infection (79, 80), sex differences have not yet been examined and may reflect a sex-specific fetal placental immune response to maternal SARS-CoV-2 infection. Together, these results suggest a sex-specific and, in some cases, sexually dimorphic response of placental ISGs to maternal SARS-CoV-2 infection, with up-regulation of ISGs in male placentas exposed to maternal SARS-CoV-2 infection.

### Modeling the relative impact of placental gene expression, antibody features, and disease timing and severity

**Fig. 5.**
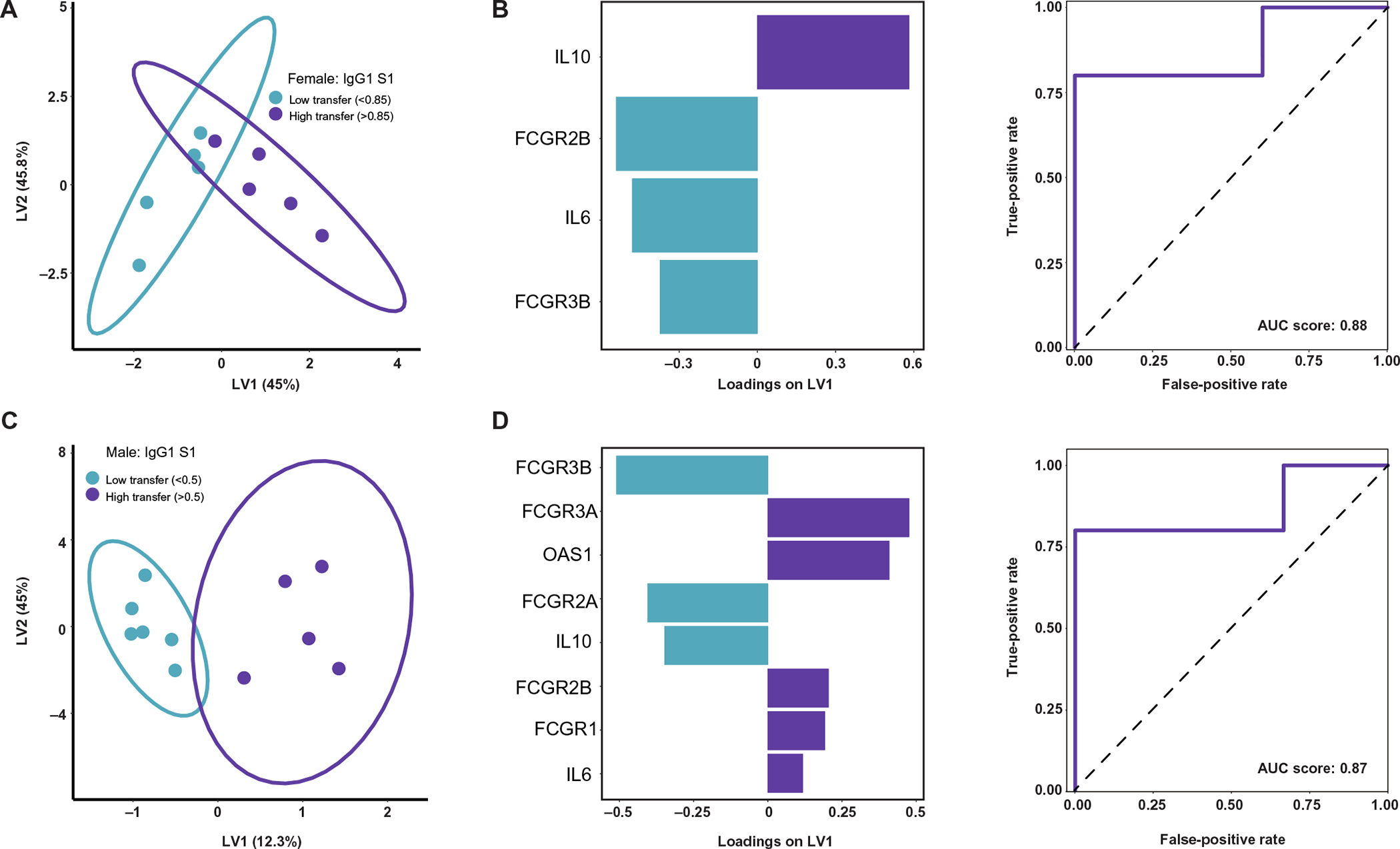
Placental gene expression affects transplacental antibody transfer. (**A**) O-PLSDA model classifying relatively low (ratio < 0.85) versus high (ratio > 0.85) IgG1 S1 transfer to female neonates based on placental gene expression. The model was built on gene expression features after LASSO feature selection. Each dot represents one mother-cord pair. (**B**) Gene expression feature loadings on the LV1 for the female model in (A). Bar color corresponds with which transfer classification (low versus high) is characterized by enriched expression of that gene, with blue bars enriched in low transfer and purple bars enriched in high transfer. The receiver operating characteristic (ROC) curve (solid purple line) for this model quantifies the accuracy in classifying IgG1 S1 transfer to female neonates after leave-one-out cross-validation. The area under the curve (AUC) score is reported. The black dashed line represents the expected performance of a random model (AUC score = 0.5). (**C**) O-PLSDA model classifying relatively low (ratio < 0.5) versus high (ratio > 0.5) IgG1 S1 transfer to male neonates based on placental gene expression. The model was built on gene expression features after LASSO feature selection. (**D**) Gene expression feature loadings on the LV1 for the male model in (C). Bar color corresponds with which transfer classification (low versus high) is characterized by enriched expression of that gene, with blue bars enriched in low transfer and purple bars enriched in high transfer, respectively. The ROC curve (solid purple line) for this model quantifies the accuracy in classifying IgG1 S1 transfer to male neonates after leave-one-out cross-validation. The AUC score is reported with black dashed line representing the expected performance of a random model (AUC score = 0.5).

Although we observed sexually dimorphic expression of individual placental genes and sex-biased transplacental antibody transfer in response to maternal SARS-CoV-2 infection, it was still unclear whether placental gene expression influenced the transfer of humoral immunity in a sex-specific manner. To investigate the relationship between fetal sex, placental gene expression, and antibody transfer, we examined whether S1-specific IgG1 transplacental transfer could be predicted by differential inflammatory, Fc receptor, and IFN-stimulated placental gene expression in male and female orthogonal partial least squares discriminant analysis (O-PLSDA) models. S1-specific IgG1 transfer was modeled because the most robust sex difference was noted in the transfer of this antibody (Fig. 1G). Given that transfer of SARS-CoV-2 specific antibodies in fetuses of both sexes was less than the expected cord:maternal ratio of greater than 1.1 to 1.5, there was no truly high or efficient transfer. Ratio thresholds for distinguishing higher and lower transfer were therefore determined on the basis of the natural distribution of the data. For female neonates, this ratio was 0.85, and for males, this ratio was 0.5 (reflective of more impaired transfer in males). To investigate whether multivariate analysis could identify gene signatures specific to relatively high or low transfer, we performed O-PLSDA separately on the female and male neonates (Fig. 5, A to D). To avoid overfitting the O-PLSDA model, features were reduced to the “optimal” set of nonredundant features using the least absolute shrinkage and selection operator (LASSO) feature selection (81). In both male and female pregnancies, high placental expression of *FCGR3B* was associated with lower IgG1 S1 transfer efficiency (Fig. 5, A to D). Higher placental *IL10* expression was associated with higher IgG1 S1 transfer in female pregnancies, but lower transfer efficiency in male pregnancies (Fig. 5, B and D). In male pregnancies, high expression of *OAS1* was associated with higher transfer (Fig. 5D). Overall, these O-PLSDA models support a systemic sexually dimorphic response that triggers distinct inflammatory cascades likely critical for driving different titers of antibody production in mothers.

We also examined the sex-specific impacts of other disease and birth-related factors on placental ISG and Fc receptor expression and placental antibody transfer. Using linear regression and O-PLSDA models, we did not observe an impact of time from infection, disease severity, or maternal labor status on male or female placental expression of any ISGs, proinflammatory cytokines, Fc receptors, or IgG transfer (fig. S11). In addition, although both male and female neonates born to SARS-CoV-2–positive mothers had reduced birthweight compared with sex-matched controls, birthweights were in the normal range for both groups (Table 1) and birthweight did not affect IgG antibody transfer in linear regression models (fig. S11). There were also no associations between antibody transfer and days from infection or gestational age at delivery for either male or female neonates (fig. S11). In summary, disease severity, time from infection, maternal labor status, neonatal birthweight, and gestational age at delivery do not appear to confound our findings of decreased transplacental antibody transfer in the context of a male fetus and sexually dimorphic placental gene expression in the context of maternal SARS-CoV-2 infection.

## DISCUSSION

Our results demonstrate the impact of fetal sex on the maternal and placental immune response to SARS-CoV-2 and the potential consequences for neonatal antibody-mediated immunity. We show that maternal SARS-CoV-2 infection is associated with reduced maternal SARS-CoV-2–specific IgG titers in the setting of a male fetus. SARS-CoV-2–specific placental antibody transfer to the male fetus was reduced despite up-regulation of placental Fc receptors in SARS-CoV-2–exposed male placentas; males were unable to overcome the reduced maternal titers and the highly fucosylated glycan profile of the spike protein–specific antibodies. Mirroring Fc receptor expression, placental expression of ISGs and proteins was also sexually dimorphic, with notable up-regulation noted in male placentas in the setting of maternal SARS-CoV-2 infection. Collectively, these findings provide evidence of maternal-placental-fetal immune cross-talk in the setting of maternal viral infection, with fetal sex playing a key role in modifying maternal humoral responses and placental innate and adaptive immune responses.

Epidemiologic data point to a persistent male bias in the development and severity of COVID-19 disease in adults, children, and infants (6, 8, 9, 82, 83). Male COVID-19 patients are three times more likely to require admission to intensive care units and have higher odds of death than females (84). This male-biased vulnerability to maternal SARS-CoV-2 infection mirrors the male-biased risk of mortality and morbidity across the perinatal period (1–3). Our findings of sexually dimorphic placental innate immune responses to infection, coupled with sex differences in transfer of maternal humoral immunity, may provide insight into increased vulnerability of male infants to morbidity and mortality.

Although the impact of fetal sex is not consistently evaluated in studies of placental function (85), sex-specific alterations in the placental transcriptome have been described in both normal and pathologic pregnancies (86–89). Sex differences in the placental immune response to prenatal infections and other immune stressors have been described in human and animal models (54, 90–93) but have not been examined in SARS-CoV-2 infection. Here, we report that maternal SARS-CoV-2 infection induces a sexually dimorphic placental antiviral innate immune response, with up-regulation of ISGs in male, but not female, placentas. Male-specific stimulation of placental ISGs after SARS-CoV-2 exposure is consistent with the heightened male immune responses reported in SARS-CoV-2–infected adult and pediatric cohorts (5, 6, 8–10, 42, 94). Although we did not see evidence of maternal viremia or placental, cord blood, or neonatal SARS-CoV-2 infection (28, 95), and the majority of maternal infections represent mild or moderate disease, there is still evidence of altered placental gene expression and an antiviral response in the placentas of male pregnancies. This indicates that even a mild maternal infection in the absence of placental or fetal infection has the potential to affect placental function and fetal development.

Because of their immature immune system, newborns rely on the passive transplacental transfer of maternal antibodies for initial protection against infectious pathogens (15, 18, 96). Although previous reports in adults have noted sex differences in the production of SARS-CoV-2–specific antibodies (42, 97), sex-biased maternal production and transplacental transfer of SARS-CoV-2–specific antibodies have not been well described. We previously reported impaired placental transfer of maternal SARS-CoV-2–specific antibodies in the setting of maternal COVID-19 (28, 29). Although there are known sex differences in adult antibody production in response to SARS-CoV-2 infection (42, 94), little is known about sex differences in maternal titers or transplacental antibody transfer (98, 99) in the setting of maternal SARS-CoV-2 infection. Our finding of decreased maternal antibody titers against all measured SARS-CoV-2–specific antigens (S, S1, S2, RBD, and N) when the fetus was male versus female was a difference not observed for influenza- or pertussis-specific antibodies. Reduced maternal SARS-CoV-2–specific IgG titer in male pregnancies was undoubtedly a driver of the reduced transplacental transfer noted in male fetuses (15). This finding of impaired placental transfer of SARS-CoV-2–specific antibodies, more pronounced in males, is consistent with the male-specific reduction of placental transfer of maternal IgG reported in a nonhuman primate model of maternal stress (98). Reduced maternal antibody titers in the setting of a male fetus were likely attributable to suppressed maternal proinflammatory responses in the setting of a male fetus, which have been described in prior studies and may function to improve tolerance of the fetal allograft (13, 14). The direct correlation between proinflammatory response and increased antibody production noted in COVID-19 infection (66, 67) suggests that blunted maternal inflammatory responses in the setting of a male fetus may limit maternal antibody production in the setting of acute infection. Whether the male-biased impairment in placental SARS-CoV-2–specific antibody transfer renders male infants more vulnerable to early-life SARS-CoV-2 infection remains unclear, as the amount of antibody necessary for protection against SARS-CoV-2 infection is unknown and there are few sex-disaggregated reports of neonatal (100, 101) or infant infection (8).

Although the up-regulation of Fc receptor expression in male placentas may represent a compensatory placental response driven by reduced maternal antibody titer and transplacental transfer of SARS-CoV-2 antibodies (22, 102, 103), this response was likely reinforced by the increased IFN signaling in males versus females. IFN-stimulated signaling may affect placental antibody transfer via alteration in Fc receptor expression and function (104–106); for example, type I IFN signaling is known to up-regulate Fcγ receptor expression on monocytes (107). Hofbauer cells, tissue-resident macrophages of the placenta, express FcγRI, FcγRII, and FcγRIII (23). The male-specific Hofbauer cell hyperplasia in placentas exposed to maternal SARS-CoV-2 infection could therefore also be contributing to increased placental FcγRI and FcγRIII expression in males.

Although the low maternal antibody titers in male pregnancies may have driven a compensatory up-regulation of Fc receptors in the male placenta, the up-regulation of FcγRIII and colocalization of FcγRIII with FcRn in the male placenta likely impeded placental transfer of SARS-CoV-2–specific antibodies, given their distinct Fc-glycan profile. Our Fc-glycan analysis demonstrated that SARS-CoV-2–specific antibodies were highly fucosylated in both male and female pregnancies, a posttranslational modification that lowers antibody affinity for FcγRIII (69–71). The male-specific placental increase in FcγRIII expression and colocalization of FcγRIII with FcRn might therefore present an additional impediment to transferring the already-low maternal titers of SARS-CoV-2–specific antibodies to the fetus. Males instead preferentially transferred bisected (afucosylated) and agalactosylated (G0), afucosylated spike protein–specific antibodies, as afucosylated antibodies are more easily transferred by FcγRIII. Given the inflammatory nature of G0 and B antibodies (72, 108, 109), their preferential transfer might promote a more inflammatory immune response in male fetuses.

Innate immune sensing of SARS-CoV-2 involves the activation of type I and type III IFNs and up-regulation of ISGs in target cells (110). Given the relative paucity of SARS-CoV-2 placental infection (28) in comparison to other pandemic infections such as Zika virus (ZIKV) (111), the increased ISG production and up-regulated *IL10* expression in exposed male placentas may be a protective mechanism to prevent direct placental infection and pathology. High IFN concentrations during pregnancy have proven protective against placental herpes simplex virus infection (112), and type III IFNs impair ZIKV transplacental transmission (113). Induction of ISGs is likely not universally protective, however. Whereas type III IFNs primarily serve a barrier defense role, type I and type II IFNs can serve a more classical immune activating or inflammatory role (31, 45). Animal models of viral infection in pregnancy implicate type I and type II IFNs and ISGs in impaired placental development and fetal growth restriction (46, 50, 51, 114), conditions that can have both short- and long-term impact on fetal and offspring health. We demonstrated increased expression of IFN-γ, initiator of type II IFN signaling, in male SARS-CoV-2–exposed placentas. IFN-γ and the type II IFN response have been implicated in placental spiral artery remodeling and may mediate fetal growth restriction and fetal demise in malarial and *Toxoplasma gondii* infection in pregnancy (50, 51, 115, 116). A transcriptomic analysis of SARS-CoV-2 response genes demonstrated that IFN-γ was an upstream regulator of host viral response in the setting of SARS-CoV-2 infection (117), with higher IFN-γ abundance associated with increased risk for SARS-CoV-2 viral entry (52) and increased mortality in moderate and severe COVID-19 illness (53). Thus, it remains unclear whether the male-specific up-regulation of ISGs described here is potentially beneficial (protection from viral infection) versus harmful (increased placental inflammation, increased risk for fetal growth restriction, or poor placental function). It was noteworthy that female placentas from SARS-CoV-2–negative control pregnancies generally had higher expression of ISGs and proteins than did male SARS-CoV-2–negative placentas. The potential for a baseline female “antiviral placental advantage” is consistent with the established increased vulnerability of the male fetus to in utero insults, including viral and bacterial infection (92, 118), and observed sex differences in baseline innate immunity described in nonplacental cells and tissues (12, 119). These findings highlight the necessity of future studies assessing baseline differences in male and female placental immune responses. The long-term consequences of SARS-CoV-2–associated placental induction of type I, II, and III IFN responses for fetal development and in utero programming of later-life metabolic and neurodevelopmental outcomes remains to be determined.

A limitation of our study is the infection of participants primarily in the third trimester, because these samples were collected during the initial wave of the SARS-CoV-2 pandemic in Boston. Whether maternal SARS-CoV-2 infection in the first and second trimester alters ISG and Fc receptor expression, and how such altered expression might durably affect placental immune function, is a question that remains to be answered in future studies. It remains unclear whether the reduced SARS-CoV-2–specific maternal antibody titers, highly fucosylated glycan profile of spike protein–specific antibodies, and attenuated male-specific transplacental antibody transfer are unique to SARS-CoV-2 biology or whether these phenomena instead reflect a common response to de novo infection during pregnancy. Future studies should assess the effect of fetal sex on maternal SARS-CoV-2 antibody titers and transplacental transfer in women infected before pregnancy and the effect of fetal sex on maternal antibody responses to other de novo infections during pregnancy. In addition, although we found no association between disease severity and placental gene expression or antibody transfer, such examinations were limited by the relatively small number of women with severe or critical illness. Although our results demonstrate male-specific up-regulation of type I and II IFNs (IFN-α and IFN-γ) and ISGs and proteins downstream of type I–III signaling cascades, this study did not assess protein expression of type III IFN-λ. Last, although our regression models did not find time from infection to delivery to be a substantial contributor to the antibody transfer ratios, we cannot entirely rule out any contribution of timing of maternal infection to the reduced antibody transfer noted in males. However, our robust sexually dimorphic gene and protein expression results, with up-regulation of both placental ISGs and Fc receptors in males, demonstrate placental factors are a stronger driver of antibody transfer than any time-from-infection effect.

In conclusion, our comprehensive evaluation of the impact of fetal sex on placental gene expression and transplacental antibody transfer in maternal SARS-CoV-2 infection provides insight into sexually dimorphic or sex-specific placental innate and adaptive immune responses to maternal SARS-CoV-2 infection. The increased impact of maternal SARS-CoV-2 infection on male placental and neonatal immunity highlights the importance of evaluating fetal sex in future studies of placental pathology and infant outcomes in SARS-CoV-2, as well as the critical importance of disaggregating sex data in follow-up studies of offspring neurodevelopmental and metabolic outcomes. These findings may have broader implications for understanding placental immune response, male vulnerability, and passive transfer of maternal antibody in other viral infections. Studies investigating SARS-CoV-2 vaccine safety and efficacy in pregnant women should also evaluate placental immune response and antibody transfer effects, in addition to neonatal infection rates, and report these data in a sex-disaggregated fashion (120).

## MATERIALS AND METHODS

### Study design

The primary objective of this study was to assess maternal-fetal antibody transfer, viral-induced placental IFN responses, and the impact of fetal sex in pregnant humans infected with SARS-CoV-2. No sample size calculation was performed as expected effect sizes were not known. Upon enrollment, collected samples were assigned ordinal deidentified sample identification numbers not specific to disease status (SARS-CoV-2 positive versus negative). Pregnant women at two tertiary care centers in Boston, Massachusetts, were approached for enrollment in a COVID-19 pregnancy biorepository study starting 2 April 2020 (Massachusetts General Hospital and Brigham and Women’s Hospital; Mass General Brigham IRB approval no. 2020P000804). All participants provided written informed consent (virtual). Pregnant women were eligible for inclusion if they were (i) 18 years or older, (ii) able to provide informed consent or had a health care proxy to do so, and (iii) diagnosed with, or at risk for, SARS-CoV-2 infection. Because of wide community spread in Massachusetts during the study period (121), all pregnant women presenting for hospital care were deemed “at risk” for SARS-CoV-2 infection. Universal COVID-19 screening of all patients admitted to the labor and delivery units by nasopharyngeal reverse transcription polymerase chain reaction (RT-PCR) testing was performed (initiated 16 April 2020). Maternal SARS-CoV-2 positivity was defined by a positive clinical nasopharyngeal RT-PCR test result. The number of replicates is detailed in the figure legends for individual experiments.

### Maternal and cord blood collection and processing

Sample collection protocols have been described in a previous publication (122). Briefly, blood from pregnant women was collected at delivery by venipuncture into EDTA tubes. Umbilical cord blood was collected immediately after delivery. The umbilical cord was wiped clean, and blood was drawn directly from the vein using a syringe and transferred to EDTA vacutainer tubes. Blood was centrifuged at 1000*g* for 10 min at room temperature. Plasma was aliquoted into cryogenic vials and stored at −80°C.

### Placental sampling and processing

Fetal side biopsies (0.5 cm^3^ per biopsy) were collected immediately after delivery. Biopsies were taken from two separate locations at least 4 cm from the cord insertion avoiding surface vasculature, edges, and areas of gross pathology. Fetal membranes were dissected from the fetal and maternal surfaces and not included in the sample. Biopsies were then dissected in half, serially washed in Dulbecco’s phosphate-buffered saline, submerged in 5 volumes of RNAlater (Invitrogen), and stored at least 24 hours at 4°C. Excess RNAlater was removed by blotting on Kimwipes, and biopsies were subdivided into 50-mg segments, placed into cryovials, flash frozen in liquid nitrogen, and stored at −80°C. For histopathological examination, placentas were fixed in formalin, weighed, examined grossly, and routine sections were taken for histopathologic diagnoses per the Amsterdam Consensus Criteria recommendations. Additional details on placental sectioning and staining are provided in the Supplementary Materials and in Methods. Placental homogenates were used to determine concentrations of the immune proteins using the Human Inflammation 20-Plex ProcartaPlex Panel (Thermo Fisher Scientific) according to the manufacturer’s instructions. Immune proteins included IFN-α, IFN-γ, IL-1α, IL-1β, IL-4, IL-6, IL-8, IL-12p70, IL-13, IL-17A, TNF-α, CXCL10, CCL2, CCL3, CCL4, CD62E, and CD62P, and protein was quantified using ProcartaPlex Analysis software (Thermo Fisher Scientific).

### Statistical analyses

All multivariate analyses were performed using R (version 4.0.0). Multivariate analyses included reverse transcription quantitative PCR (RT-qPCR) and Luminex antibody measurements. Missing data measurements were imputed using the *k*-nearest neighbor algorithm. Features were then centered and scaled to unit variance. O-PLSDA was performed to classify data on the basis of relative antibody transfer. Before building the O-PLSDA models, LASSO feature regularization and variable selection was performed, resulting in a reduced set of features that were consistently included in at least 80 of the 100 LASSO models. O-PLSDA models were built using the R “ropls” Bioconductor package (orthI = 1 and PredI = 1). Models were validated by evaluating the mean accuracy score after 100 trials of fivefold coefficient of variation (CV). Accuracy scores for analogous models with permutated labels and random features were also reported for comparison.

Statistical tests used and statistical significance are reported in the figure legends. Statistical analyses were performed using GraphPad Prism 9 and R (version 4.0.0). Significant differences in participant characteristics were evaluated using Kruskal-Wallis tests for continuous variables and chi-square or Fisher’s exact tests for categorical variables. Gene expression measurements, immunoblot measurements, and immunohistochemistry staining comparing maternal SARS-CoV-2 exposure status and fetal sex were evaluated by two-way analysis of variance (two-way ANOVA) with Bonferroni’s post hoc analysis. Kruskal-Wallis test followed by Dunn’s post hoc test was used to determine significant differences between transfer ratios. Wilcoxon matched-pairs signed rank test was performed to determine significant differences between paired maternal-cord blood samples. All linear regression models were built in R (version 4.0.0) using the “stats” package. False discovery rate (FDR) multiple comparisons correction was performed for fig. S6 using a corrected *P* value of 0.05 as the cutoff for significance.

## Supplementary Material

Data

Supplementary Material

## Figures and Tables

**Table 1.** Demographic and clinical characteristics of study cohort by fetal sex and maternal SARS-CoV-2 status.

All (68)		Female		Male		*P* ^†^
		SARS-CoV-2 negative (15)	SARS-CoV-2 positive (19)*	SARS-CoV-2 negative (15)	SARS-CoV-2 positive (19)*	
Maternal age, years	33 [28–38]	33 [30–36]	34 [30–40]	30 [27–37]	31 [26–36]	0.34
Parity, *n*	1 [0–2]	1 [0–2]	1 [0–2]	1 [0–2]	1 [0–2]	0.34
Race, *n* (%)						0.19
White	40 (59)	11 (73)	8 (42)	12 (80)	9 (59)	
Black	5 (7)	1 (7)	3 (16)	0 (0)	1 (7)	
Asian	1 (1)	0 (0)	0 (0)	1 (7)	0 (0)	
Other	12 (18)	3 (20)	4 (21)	1 (7)	4 (8)	
Not reported	10 (15)	0 (0)	4 (21)	1 (7)	5 (15)	
Ethnicity, *n* (%)						0.01
Hispanic	32 (47)	4 (27)	11 (58)	2 (13)	15 (79)	
Non-Hispanic	33 (48)	10 (67)	7 (37)	12 (80)	4 (21)	
Not reported	3 (4)	1 (7)	1 (5)	1 (7)	0 (0)	
Chronic hypertension, *n* (%)	0 (0)	0 (0)	0 (0)	0 (0)	0 (0)	N/A
DM/GDM, *n* (%)	6 (9)	1 (7)	3 (16)	1 (7)	1 (5)	0.67
BMI ≥ 30 kg/m^2^, *n* (%)	20 (29)	2 (13)	7 (37)	3 (20)	8 (42)	0.21
Prepregnancy BMI, kg/m^2^	27.4 [21.5–30.8]	25.8 [21.6–29.4]	28.5 [26.5–32.7]	21.5 [20.3–29.8]	29.5 [22.3–32.0]	0.06
GA at delivery, weeks	39.2 [38.6–40.3]	39.3 [39.0–39.4]	39.3 [38.4–40.1]	39.1 [39.0–41.1]	39.0 [35.3–40.3]	0.09
Any labor, *n* (%)	52 (76)	8 (53)	16 (84)	11 (73)	17 (89)	0.07
Neonatal birthweight, *g*	3283 [3005–3590]	3400 [3060–3590]	3255 [2920–3340]	3435 [3260–3730]	3115 [2615–3590]	0.06
GA at positive SARS-CoV-2 test, weeks	38.7 [35.6–39.6]	N/A	36.3 [32.4–39.4]	N/A	36.3 [32.4–39.5]	0.90
COVID-19 disease severity at diagnosis^‡^, *n*						0.82
Asymptomatic	16 (42)	N/A	8 (42)	N/A	8 (42)	
Mild/moderate	19 (50)	N/A	9 (47)	N/A	10 (53)	
Severe/critical	3 (8)	N/A	2 (11)	N/A	1 (5)	
Time between SARS-CoV-2 symptom onset and delivery, days	36.5 [18–57]	N/A	44 [28–64]	N/A	32 [8–51]	0.18

DM/GDM, diabetes mellitus/gestational diabetes mellitus; BMI, body mass index; GA, gestational age; N/A, not applicable.

* SARS-CoV-2 status determined by nasopharyngeal RT-PCR at time of sample collection. If a participant was SARS-CoV-2 positive at any time in pregnancy, then she was included in “SARS-CoV-2 positive” category.

† Significant differences between groups were determined using chi-square test for categorical variables, and Kruskal-Wallis test for continuous variables presented as median [interquartile range].

‡ Disease severity classifications are based on published National Institutes of Health criteria.
