# Supplementary material for: Maternal SARS-CoV-2 infection elicits sexually dimorphic placental immune responses
Source: Sci Transl Med. Author manuscript; Available in PMC 2022 Jan 24. (PMC8784281; doi:10.1126/scitranslmed.abi7428)
Supplement: Supplementary Material [file NIHMS1758129-supplement-Supplementary_Material.pdf]

**SUPPLEMENTARY MATERIALS**

# Maternal SARS-CoV-2 infection elicits sexually dimorphic placental immune responses

Evan A Bordt*, Lydia L Shook*, Caroline Atyeo*, Krista M Pullen*, Rose M De Guzman, Marie-Charlotte Meinsohn, Maeva Chauvin, Stephanie Fischinger, Laura J. Yockey, Kaitlyn James, Rosiane Lima, Lael M Yonker, Alessio Fasano, Sara Brigida, Lisa M Bebell, Drucilla J Roberts, David Pépin, Jun R Huh, Staci D Bilbo, Jonathan Z Li, Anjali Kaimal, Danny Schust, Kathryn J Gray, Douglas Lauffenburger, Galit Alter, Andrea G Edlow

*These authors contributed equally to this work

**Address correspondence to:**
Andrea G Edlow
Vincent Center for Reproductive Biology
Massachusetts General Hospital
55 Fruit Street, Thier Research Building, 903b
Ph: 617-724-0654
Email: aedlow@mgh.harvard.edu

**This PDF file includes:**

Supplementary Materials and Methods

Fig. S1. SARS-CoV-2 infected mothers with male fetuses have lower plasma titers of SARS-CoV-2-specific antibodies

Fig. S2. SARS-CoV-2 infected mothers with male fetuses demonstrate reduced placental transfer of SARS-CoV-2 antibodies compared to those with female fetuses.

Fig. S3. Maternal and cord blood titers of HA and PTN in SARS-CoV-2 infected and non-infected mothers.

Fig. S4. Transplacental transfer of endemic, chronic and other childhood vaccinatable pathogens by maternal SARS-CoV-2 status and fetal sex.

Fig. S5. Fetal sex does not affect maternal plasma titers of non-SARS-CoV-2 antibodies.

Fig. S6. No effect of maternal SARS-CoV-2 infection on expression or localization of FCγR2.

Fig. S7. Fc-glycan glycoforms in maternal bulk and SARS-CoV-2-specific antibodies.

Fig. S8. Maternal SARS-CoV-2 infection does not impact placental expression of *TNF*, *IL6*, or *CCL7*.

Fig. S9. Maternal SARS-CoV-2 infection and fetal sex do not impact placental expression of reference genes *YHWAZ* or *TOP1*.

Fig. S10. Maternal SARS-CoV-2 infection does not impact placental cytokine expression.

Fig. S11. Effect of disease severity, time since infection, neonatal birthweight, gestational age, and labor status on gene expression and antibody transfer.

Table S1. Demographic and clinical characteristics of participants providing maternal and cord blood by fetal sex and maternal SARS-CoV-2 status.

Table S2. Timing of Influenza and pertussis vaccination relative to maternal titers drawn at delivery hospitalization.

Table S3. 2-way ANOVA analysis of Fc receptor gene expression, immunoblots, and immunohistochemistry.

Table S4. 2-way ANOVA analysis of inflammatory cytokine and interferon stimulated gene expression.

Table S5. Taqman gene expression assays used for qPCR.

## SUPPLEMENTARY MATERIALS AND METHODS

### Systems Serology

A multiplexed Luminex assay was used to determine relative titer of antigen-specific isotypes, subclasses, and Fc receptor (FcR) binding, as previously described *(45)*. The following antigens were used in this assay: severe acute respiratory syndrome coronavirus 2 (SARS-CoV-2) receptor binding domain (RBD, Sino Biological), SARS-CoV-2 spike (S) protein (LakePharma), SARS-CoV-2 nucleocapsid (N) protein (Aalto Bio Reagents), SARS-CoV-2 S1 (Sino Biological), SARS-CoV-2 S2 (Sino Biological), pertussis pertactin (List Reagents) and a mix of HA A/Michigan/45/2015 (H1N1), HA A/Singapore/INFIMH-16-0019/2016 (H3N2), B/Phuket/3073/2013 (Immunetech). Antigens were covalently linked to carboxyl-modified Magplex Luminex beads using Sulfo-NHS (N-hydroxysulfosuccinimide, Pierce) and ethyl dimethylaminopropyl carbodiimide hydrochloride (EDC). Antigen-coupled microspheres were blocked, washed, resuspended in phosphate-buffered saline (PBS), and stored at 4°C. Plasma (diluted 1:100 for IgG2 and 3, 1:500 for IgG1 and neonatal Fc receptor (FcRn), 1:1000 for all other FcRs) was added to the antigen-coupled microspheres to form immune complexes, and plates were incubated overnight at 4˚C, shaking at 700 rpm. The next day, plates were washed with 0.1% bovine serum albumin (BSA) and 0.02% Tween-20 in PBS. Phycoerythrin (PE)-coupled mouse anti-human detection antibodies (Southern Biotech) were used to detect antigen-specific antibody binding. Avi-Tagged FcRs (Duke Human Vaccine Institute) were biotinylated using BirA500 kit (Avidity) per manufacturer's instructions to detect FcR binding. Biotinylated FcRs were tagged with PE and added to immune complexes. Fluorescence was acquired using an Intellicyt iQue. Relative antigen-specific antibody titer and FcR binding was reported as Median Fluorescence Intensity (MFI).

### RNA extraction

Placental tissue was placed in Trizol (100 µL/10 mg) and homogenized using a TissueTearor. The resulting suspension was then centrifuged at 12,000 *x* g for 10 minutes after which the pellet was discarded. Chloroform was added to supernatant at a ratio of 100 µL chloroform/50 mg tissue. Tubes were shaken vigorously for 15

seconds, allowed to stand at room temperature for 10 minutes, and then centrifuged at 12,000 *x* g for 15 minutes. The aqueous phase was collected and the remainder of the RNA extraction procedure was performed using an RNeasy Mini Kit with on-column DNase I treatment (Qiagen) according to manufacturer instructions. RNA quantity and purity were assessed using a NanoDrop 2000 Spectrophotometer (Thermo Fisher Scientific).

**cDNA synthesis and quantitative polymerase chain reaction (qPCR)**

cDNA synthesis was performed using the iScript cDNA Synthesis Kit (Bio-Rad) according to manufacturer instructions. Briefly, 800ng of RNA was mixed with iScript Reaction Mix, iScript Reverse Transcriptase, and nuclease-free water. No template control and No reverse transcriptase (RT) controls were prepared. All samples were primed at 25°C for 5 minutes, heated to 46°C for 20 minutes, and RT was inactivated at 95°C for 1 minute using a MiniAmp Plus Thermal Cycler (Thermo Fisher Scientific). qPCR was then performed using Taqman gene expression assays on a QuantStudio 5 Real-Time PCR System (Thermo Fisher Scientific). Gene expression assays used are listed in table S5. Gene expression was normalized to the placental reference gene Tyrosine 3-Monooxygenase/Tryptophan 5-Monooxygenase Activation Protein Zeta (*YWHAZ*) and expressed relative to female coronavirus disease 2019 (COVID-19) negative samples to yield a relative quantity value ($2^{-\Delta\Delta Ct}$). Importantly, the average values for female negative sample used for normalization was obtained using all female negative samples. That is, every experimental sample would have four values, as each experimental sample had four placental biopsies. Neither fetal sex or maternal SARS-CoV-2 infection altered expression of the reference gene *YWHAZ* or another common placental reference gene *TOP1*. All genes of interest were probed using gene expression assays with a dye of FAM-MGB, and *YWHAZ* and *TOP1* were assessed using gene expression assays with a dye of VIC_PL. Two separate biopsies were each run in technical duplicate, and the average of biological replicates were then used to obtain final reported $2^{-\Delta\Delta Ct}$ values.

**Protein extraction and quantification**

Placental tissue was placed in RIPA Lysis and Extraction Buffer (Thermo Fisher Scientific #89901) containing Halt Protease and Phosphatase Inhibitor Cocktail (Thermo Fisher Scientific #78443). Samples were homogenized using a TissueTearor. The resulting mixture was centrifuged at 10,000 rpm for 10 minutes at 4°C and supernatant was kept for protein quantification. Protein was quantified using Pierce BCA Protein Assay Kit per the manufacturer's instructions (Thermo Fisher Scientific #23225).

**Immunoblotting**

50 µg of protein was prepared in Protein Sample Loading Buffer (Li-Cor Biosciences) with 5 mM dithiothreitol (DTT), boiled for 10 minutes at 95°C, loaded on Mini-PROTEAN 4-20% TGX Stain-Free Precast Gels (Bio-Rad), and run at 200V for 30 minutes, after which they were transferred onto low fluorescence polyvinylidene fluoride (PVDF) membrane using a Trans-Blot Turbo Transfer System (Bio-Rad). Blots were then washed three times for 10 minutes in TBS (20X stock from Thermo Fisher diluted 1:20 in water) followed by imaging of Stain Free total protein with a ChemiDoc MP System (Bio-Rad). After stain free imaging, blots were washed three times for 10 min in TBS, followed by blocking in Intercept (TBS) Blocking Buffer (Li-Cor Biosciences) for 1 hour. Primary antibodies were then incubated in Intercept T20 (TBS) Antibody Diluent (Li-Cor Biosciences) overnight at 4°C. The next morning, membranes were washed six times for 10 minutes in TBS-T (20X stock from Thermo Fisher diluted 1:20 in water), followed by incubation with secondary antibodies at 1:15,000 dilution and hFAB Rhodamine Anti-Tubulin (Bio-Rad) at 1:4000 dilution for 1 hour in Intercept T20 (TBS) Antibody Diluent (Li-Cor Biosciences). Finally, blots were washed six times for 10 minutes in TBS-T, after which they briefly rinsed in and then placed in TBS. Fluorescence was then imaged with a ChemiDoc MP System (Bio-Rad) and quantified using Image Lab Software (Bio-Rad). Data are presented as volume of bands for protein of interest relative to the volume of bands contained in the total protein stain-free image.

**Fc glycan analysis**

Spike protein (Lake Pharma) was biotinylated and coupled to streptavidin magnetic beads (New England Biolabs, NEB). Cord and maternal plasma was heat-inactivated and spike protein-specific antibodies were isolated by incubating cord or maternal plasma with spike-coupled magnetic beads. The spike protein-specific antibody-bead complexes were incubated with IDEZ (NEB) for 1 hour at room temperature to cleave the Fc from spike protein-specific antibodies. The isolated Fc was then incubated with PNGase to remove the glycan and glycans were labelled with APTS per manufacturer's instructions (Glycan Assure APTS kit, Thermo Fisher Scientific). Glycans were analyzed using a 3500xL genetic analyzer (Applied Biosystems). The relative frequency of each glycoform was determined using the GlycanAssure Software.

**Immunofluorescence (IF)**

For co-labeling IF experiments, placenta tissue sections were rehydrated in an alcohol series after deparaffinization in xylene. Antigen retrieval was performed by boiling in 10 mM sodium citrate (pH 6.0) for 30 minutes and cooled at room temperature before blocking for 15 minutes with background sniper (Biocare Medical). Samples were then incubated in primary antibodies diluted in 5% BSA for 1.5 hours at room temperature (Placental Alkaline Phosphatase (PLAP), Abcam ab212383, 1:1000; Neonatal Fc Receptor (FcRn), Abcam ab193148, 1:100; CD16, Leica NCL-L-CD16, 1:100; CD32, R&D AF1330, 10μg/ml; CD64, Origene TA506331, 1:100). The slides were washed in PBS Tween 0.1% and incubated in fluorescently conjugated secondary antibodies at 1:400 in 5% BSA. Secondary antibodies used included Goat anti-Mouse IgG2a Alexa Fluor 546 (ThermoFisher Scientific A-21133), Goat anti-mouse IgG2b Alexa Fluor 488 (ThermoFisher Scientific A-21141), Goat anti-rabbit Alexa Fluor 594 (Abcam ab150080), Goat anti-mouse IgG Alexa Fluor 647 (BioLegend 405322), Donkey anti-goat (Abcam ab150135 Finally, the slides were treated with Vector True View to eliminate red blood cells and background (Vector True View, SP8400-15, Vector Laboratories), treated with 4′,6-diamidino-2-phenylindole (DAPI, ThermoFisher Scientific D13060) and cover-slipped with vectashield mounting medium (Vector Laboratories - H-1000).

CellProfiler software (PMID:29969450) was used to quantify CD16/FcRn, CD32/FcRn, and CD64/FcRn colocalization as well as their respective intensities. Briefly, RGB pictures were converted to gray and placental villi selected to avoid measuring background. Following background removal, the pictures were filtered using the function CorrectIlluminationCalculate and aligned to evaluate colocalization. Finally, CD16, CD32 or CD64 and FcRn intensities in the placental villi previously delimitated to quantify colocalization.

CellProlifer software was similarly used to quantify immunohistochemistry for and CD163. Following isolation of each dye compound (3,3'-Diaminobenzidine (DAB) and Hematoxylin), the placental villi were manually isolated and the mean intensity of DAB signal quantified.

## SUPPLEMENTARY FIGURES AND TABLES

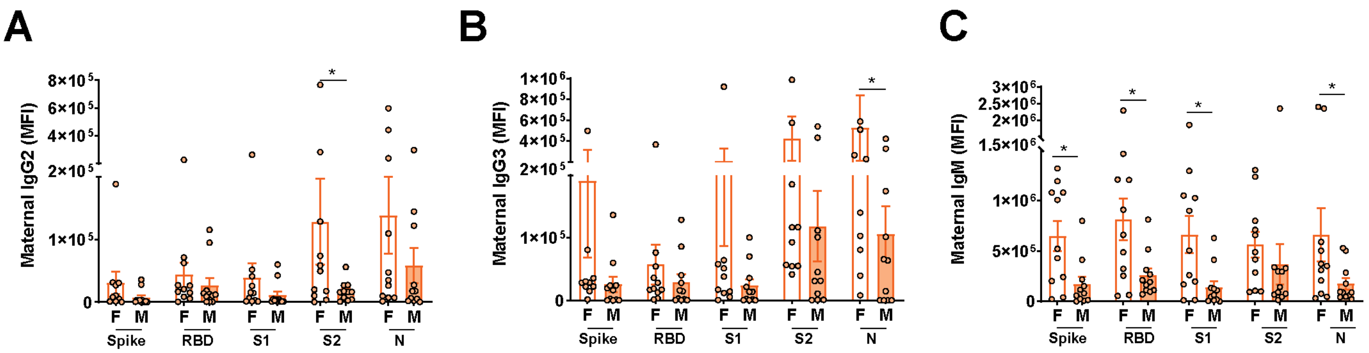


**Fig. S1. SARS-CoV-2 infected mothers with male fetuses have lower plasma titers of SARS-CoV-2-specific antibodies.**
(**A to C**) Plots show maternal spike protein-, RBD-, S1-, S2-, and N protein-specific maternal blood IgG2 (A), IgG3 (B), and IgM (C) titers as median fluorescence intensity (MFI). Female neonates of mothers with SARS-CoV-2 are shown as white bars with an orange border and male neonates are shown as orange shaded bars with orange border. Differences across groups were assessed by two-way ANOVA followed by post-hoc analyses. Two-way ANOVA demonstrated a main effect of fetal/neonatal sex on maternal IgG1 titers (IgG2 $p = 0.003$ ; IgG3 $p = 0.011$ ; IgM $p < 0.0001$). *$p < 0.05$. Data are presented as mean ± SEM.

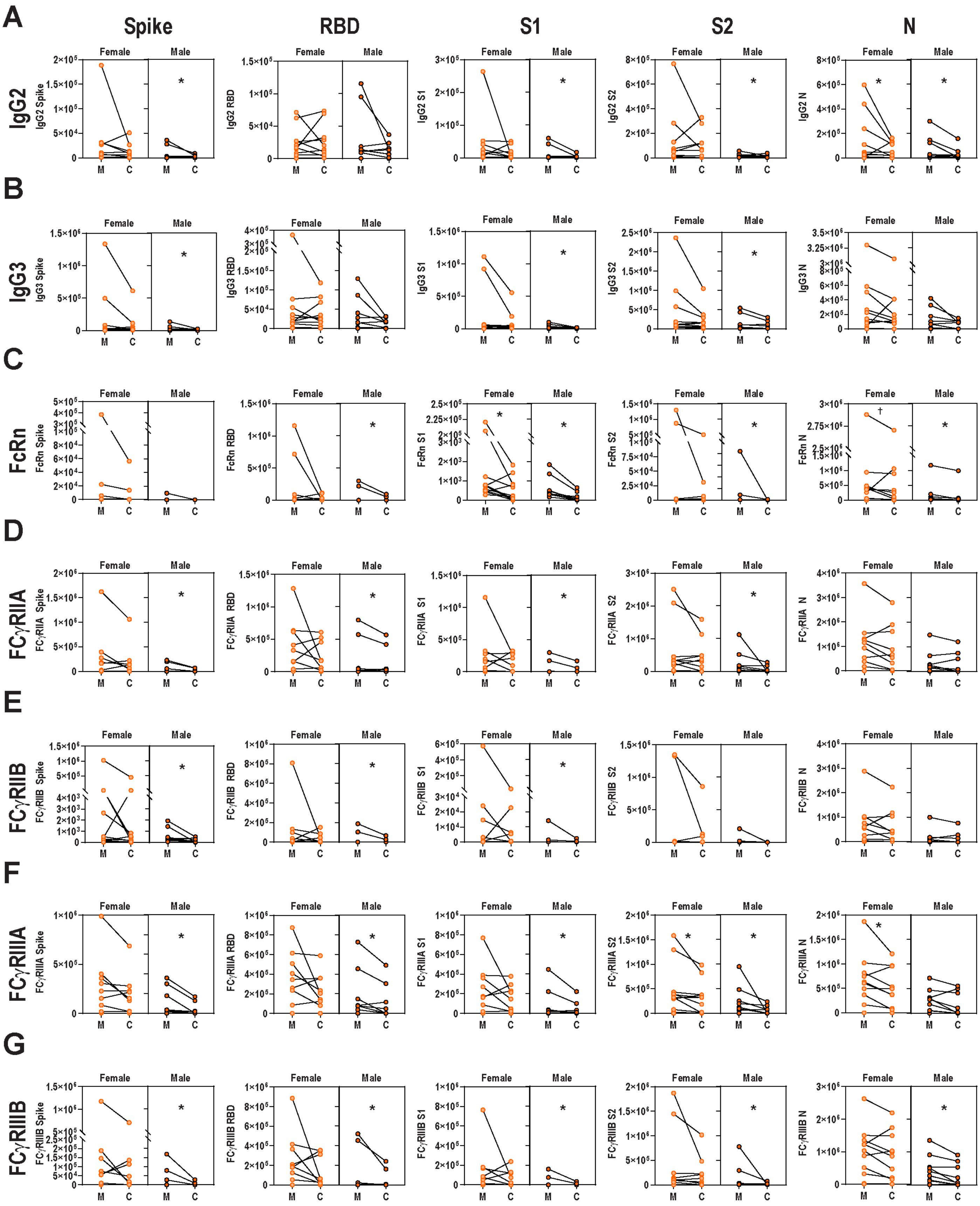
A
B
C
D
E
F
G
Spike
RBD
S1
S2
N
IgG2
IgG3
FcRn
FCγRIIA
FCγRIIB
FCγRIIIA
FCγRIIIB
Female
Male
M
C
*
†
IgG2 Spike
IgG2 RBD
IgG2 S1
IgG2 S2
IgG2 N
IgG3 Spike
IgG3 RBD
IgG3 S1
IgG3 S2
IgG3 N
FcRn Spike
FcRn RBD
FcRn S1
FcRn S2
FcRn N
FCγRIIA Spike
FCγRIIA RBD
FCγRIIA S1
FCγRIIA S2
FCγRIIA N
FCγRIIB Spike
FCγRIIB RBD
FCγRIIB S1
FCγRIIB S2
FCγRIIB N
FCγRIIIA Spike
FCγRIIIA RBD
FCγRIIIA S1
FCγRIIIA S2
FCγRIIIA N
FCγRIIIB Spike
FCγRIIIB RBD
FCγRIIIB S1
FCγRIIIB S2
FCγRIIIB N

**Fig. S2. SARS-CoV-2 infected mothers with male fetuses demonstrate reduced placental transfer of SARS-CoV-2 antibodies compared to those with female fetuses.**
(**A to G**) Dot plots show relative spike protein-, RBD-, S1-, S2-, and N protein-specific maternal blood (M) and cord blood (C) titers of IgG2 (A), IgG3 (B), FcRn (C), FCγRIIA(D), FCγRIIB (E), FCγRIIIA (F), and FCγRIIIB (G). Females are shown in light orange and males are shown in dark orange. Y-axis units for all plots are PBS-corrected median fluorescence intensity (MFI). Wilcoxon matched pairs signed rank test was performed to determine significance. *$p<0.05$, † $p<0.10$.

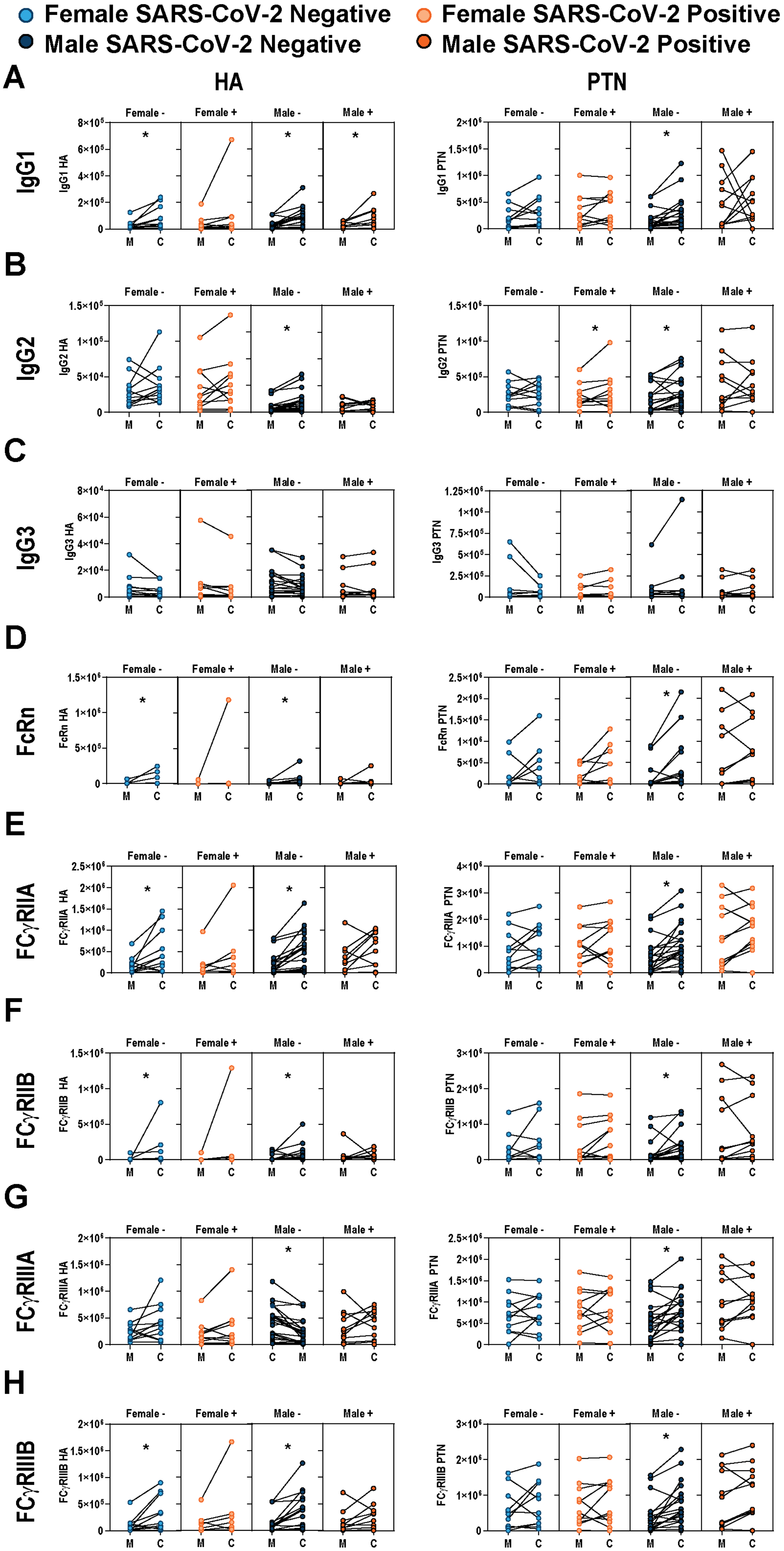

Female SARS-CoV-2 Negative
Male SARS-CoV-2 Negative
Female SARS-CoV-2 Positive
Male SARS-CoV-2 Positive
A
B
C
D
E
F
G
H
HA
PTN
IgG1
IgG2
IgG3
FcRn
FCγRIIA
FCγRIIB
FCγRIIIA
FCγRIIIB
Female -
Female +
Male -
Male +
M
C

**Fig. S3. Maternal and cord blood titers of HA and PTN in SARS-CoV-2 infected and non-infected mothers.** (**A to H**) Dot plots showing relative hemagglutinin (HA)- and pertussis (PTN)-specific maternal blood (M) and cord blood (C) titers of IgG1 (A), IgG2 (B), IgG3 (C), FcRn (D), FCγRIIA (E), FCγRIIB (F) FCγRIIIA (G), and FCγRIIIB (H). SARS-CoV-2 negative females are shown in light blue, SARS-CoV-2 positive females are shown in light orange, SARS-CoV-2 negative males are shown in dark blue, and SARS-CoV-2 positive males are shown in dark orange. Y-axis units for all plots are PBS-corrected median fluorescence intensity (MFI). Wilcoxon matched-pairs signed rank test was performed to determine significance. * $p < 0.05$.

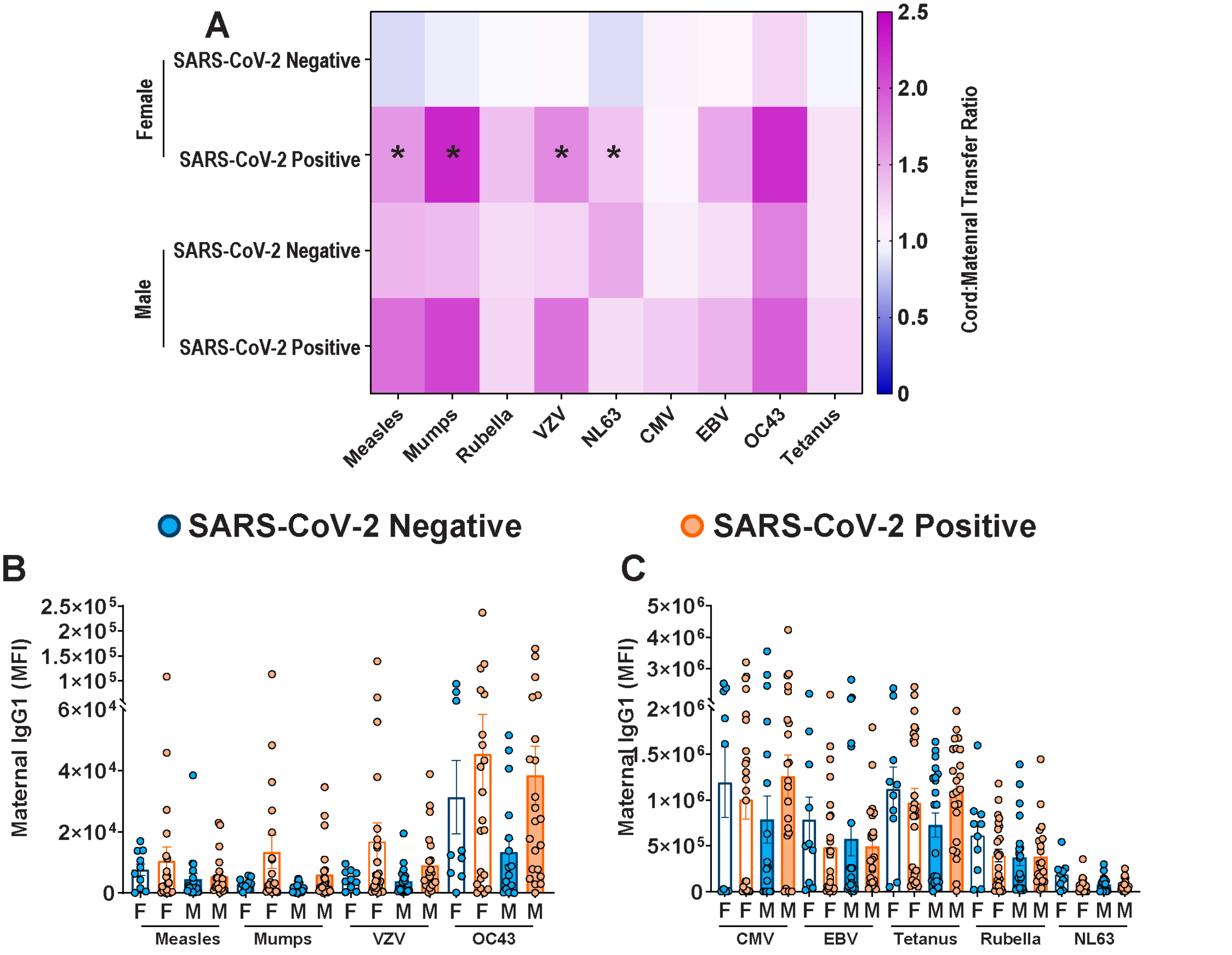


**Fig. S4. Transplacental transfer of endemic, chronic, and other childhood vaccinatable pathogens by maternal SARS-CoV-2 status and fetal sex.**

**(A)** A heatmap depicting the median PBS background-corrected cord:maternal transfer ratio of measles, mumps, rubella, varicella-zoster virus (VZV), human coronavirus NL63 (NL63), cytomegalovirus (CMV), Epstein-Barr virus (EBV), human coronavirus OC43 (OC43), and tetanus IgG1 in both SARS-CoV-2 negative (-) and SARS-CoV-2 positive (+) maternal:neonate dyads is shown. Kruskal-Wallis test followed by Dunn's post-hoc analyses were performed to determine significance. * $p < 0.05$. **(B and C)** Box-and-whisker plots show the maternal titers for IgG1 against Measles, Mumps, varicella zoster virus (VZV), and common human coronavirus OC43 (B), and cytomegalovirus (CMV), Epstein-Barr virus (EBV), Tetanus, Rubella, and common human coronavirus NL63 (C). SARS-CoV-2 negative maternal status is shown in blue (female: open bars; male: shaded bars) and SARS-CoV-2 positive maternal status is shown in orange (female: open bars; male: shaded bars). F, female; M, male. Y-axis units for all plots are PBS-corrected median fluorescence intensity (MFI). Two-way ANOVA revealed no significant effect of fetal sex or maternal SARS-CoV-2 infection status. Data in (B and C) are presented as mean ± SEM.

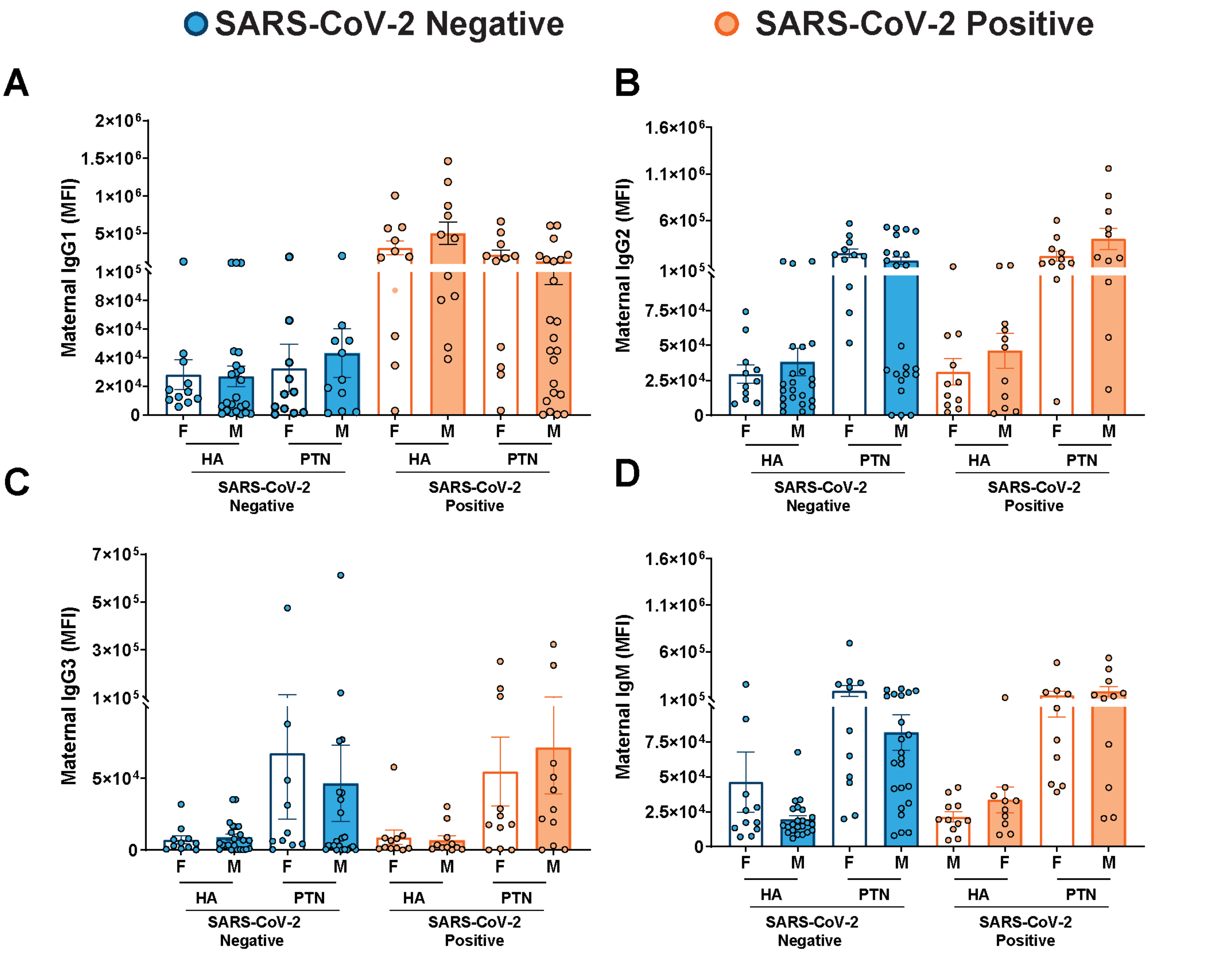


**Fig. S5. Fetal sex does not affect maternal plasma titers of non-SARS-CoV-2 antibodies.** (**A to D**) Plots showing titers of IgG1 (A), IgG2 (B), IgG3 (C), and IgM (D) against HA and PTN in maternal plasma from either SARS-CoV-2 negative or SARS-CoV-2 positive mothers. Females born to SARS-CoV-2 negative mothers are shown as white bars with blue border, females born to SARS-CoV-2 positive mothers are shown as white bars with orange border. Males born to SARS-CoV-2 negative mothers are shown as shaded blue bars with blue border, and males born to SARS-CoV-2 positive mothers are shown as orange shaded bars with orange border. Y-axis units for all plots are PBS-corrected median fluorescence intensity (MFI). Two-way ANOVA revealed no significant effect of fetal sex. Data are presented as mean ± SEM.

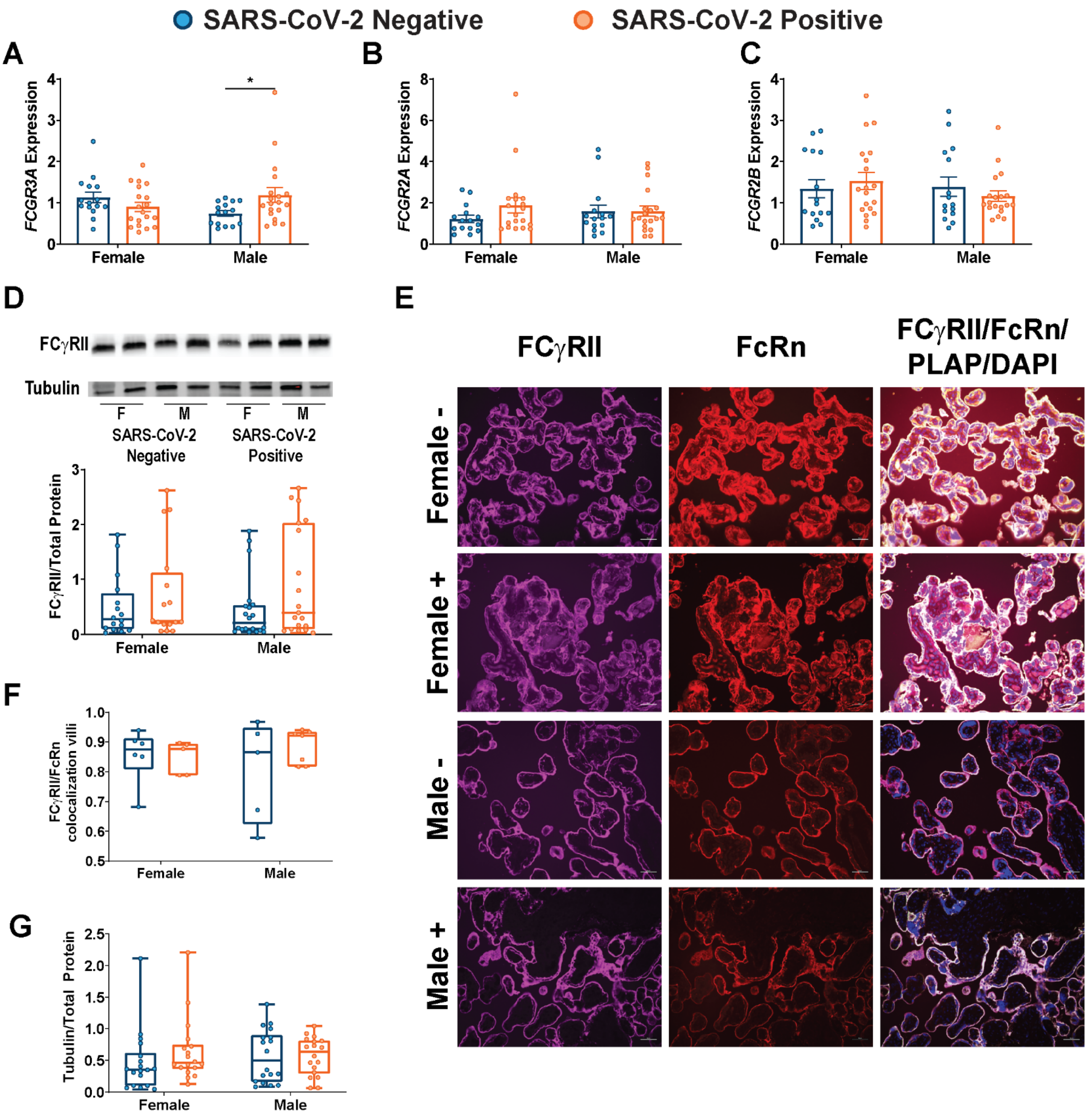


**Fig. S6. No effect of maternal SARS-CoV-2 infection on expression or localization of FCγR2.**
**(A to C)** qPCR analyses of fetal male or fetal female expression of *FCGR3A* (A), *FCGR2A* (B), and *FCGR2B* (C) in placental biopsies from SARS-CoV-2 negative (blue) or SARS-CoV-2 positive (orange) pregnancies. Data are presented as mean ± SEM Expression values shown are relative to reference gene *YWHAZ*. Differences across groups were assessed by two-way ANOVA followed by Bonferroni post-hoc analyses. * $p<0.05$.
**(D)** Representative immunoblots and quantification of FCγR2 in female or male placental biopsies from mothers testing negative (blue) or positive (orange) for SARS-CoV-2. **(E)** Placental tissue sections from SARS-

CoV-2 positive and SARS-CoV-2 negative mothers were stained for FCγR2 (purple), FcRn (red), and placental alkaline phosphatase (PLAP, green), a trophoblast marker, and DAPI (blue). Scale bars indicate 100 µm. **(F)** Box-and-whisker plots show FCγR2/FcRn co-localization in placental villi. Two-way ANOVA followed by post-hoc analyses were performed to determine significance. **(G)** Box-and-whisker plots show tubulin/total protein quantification across fetal sex and maternal SARS-CoV-2 infection status. Two-way ANOVA revealed no significant effect of fetal sex or maternal infection. For box and whisker plots in D, F, and G, box extends from $25^{th}$-$75^{th}$ percentile, the whiskers depict minimum and maximum, and horizontal line depicts the median.

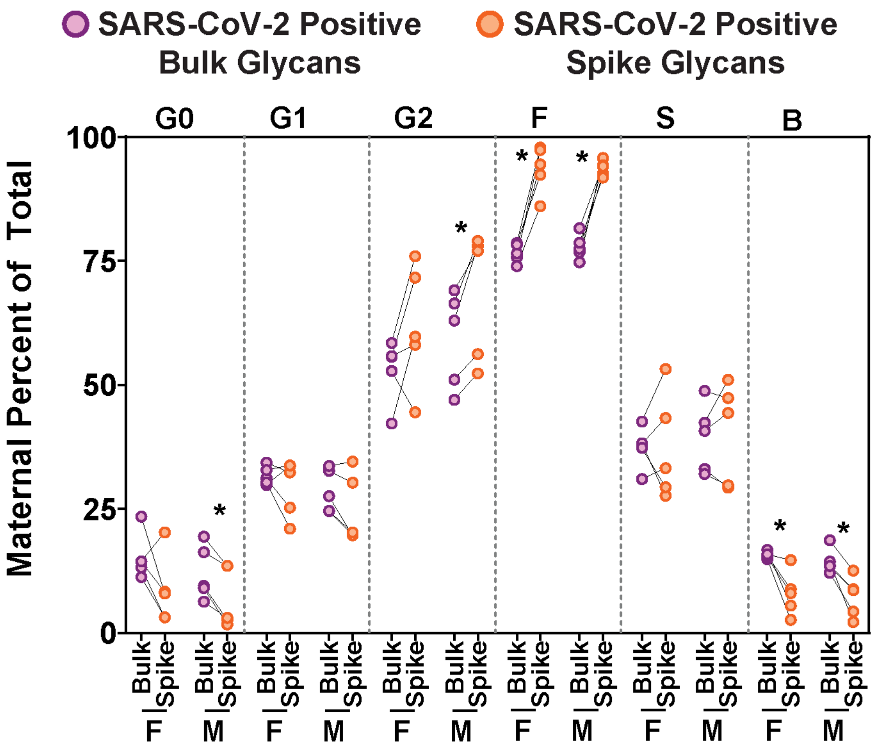


**Fig. S7. Fc-glycan glycoforms in maternal bulk and SARS-CoV-2-specific antibodies.**
Dot plots show the percentage of each glycoform in bulk maternal (purple) and spike protein-specific (orange) antibody glycan data from SARS-CoV-2-positive mothers for which matched data were available. Wilcoxon signed-rank test was performed to determine significance. * $p < 0.05$.

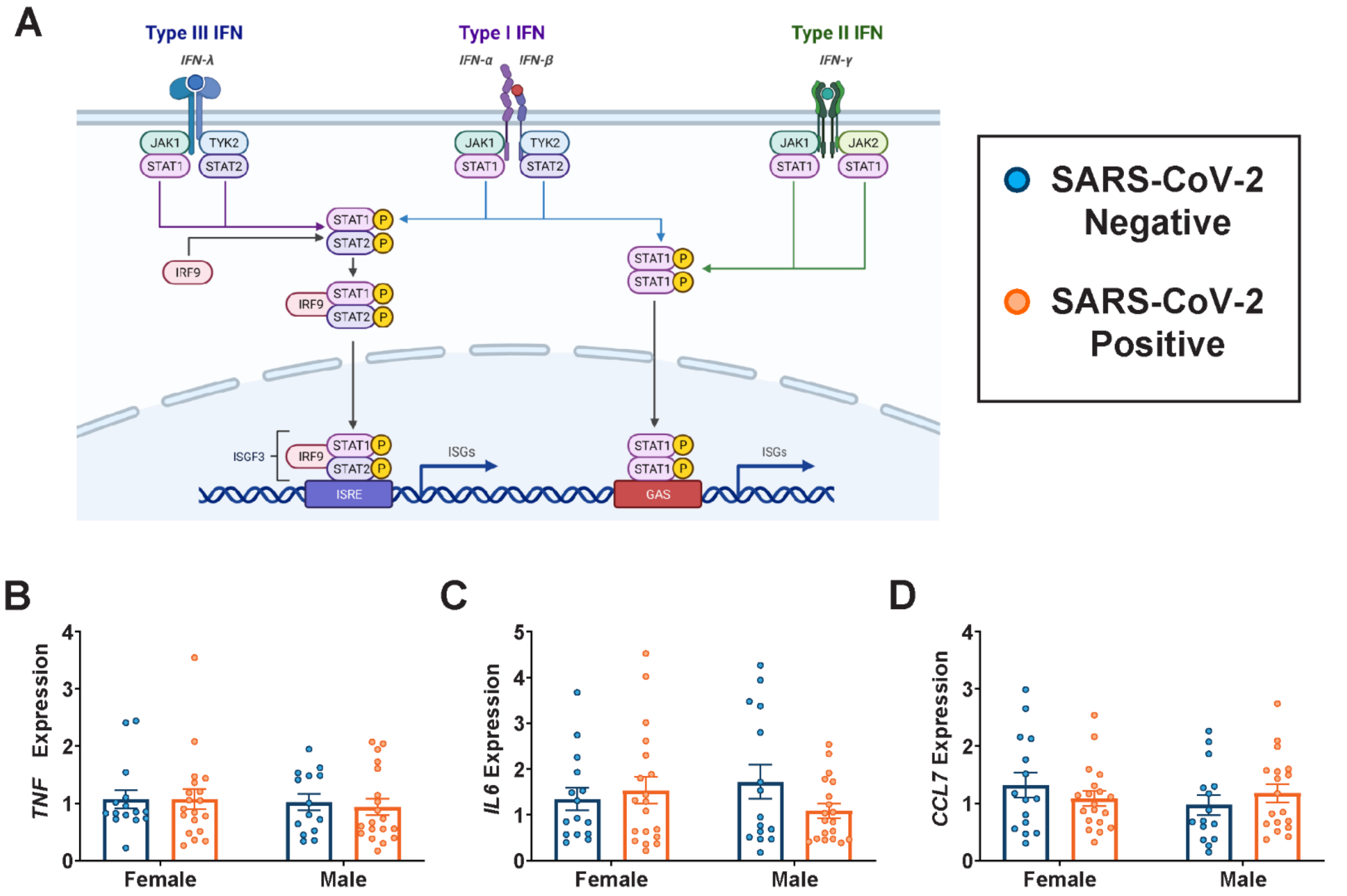


**Fig. S8. Maternal SARS-CoV-2 infection does not impact placental expression of *TNF, IL6,* or *CCL7*.**
**(A)** Interferon stimulated gene pathway diagram. Production of interferon stimulated genes (ISGs) of interest can occur through activation of Type I IFN, Type II IFN, or Type III IFN. Image created using Biorender.
**(B to D)** qPCR analyses of fetal male or fetal female expression of *TNF* (B)*, IL6* (C)*,* and *CCL7* (D) in placental biopsies from SARS-CoV-2 negative (blue) or SARS-CoV-2 positive (orange) pregnancies. Data are presented as mean ± SEM. Differences across groups were assessed by two-way ANOVA. Expression values shown are relative to reference gene *YWHAZ*.

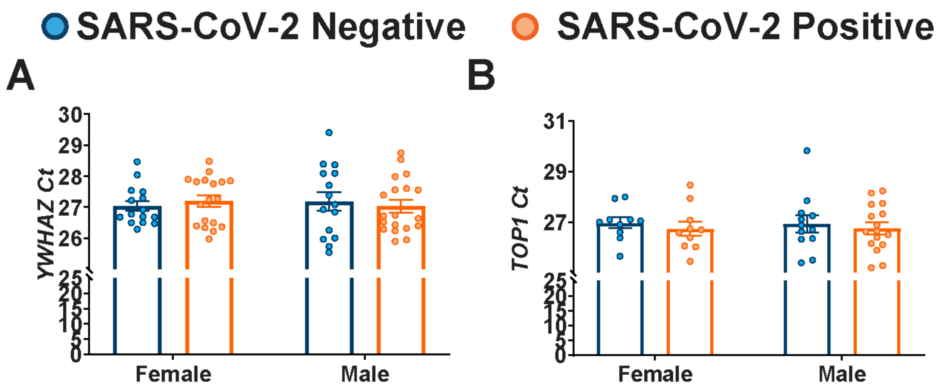


**Fig. S9. Maternal SARS-CoV-2 infection does not impact placental expression of reference genes *YWHAZ* or *TOP1*.**

**(A and B)** qPCR Ct values of fetal male or fetal female expression of *YWHAZ* (A) and *TOP1* (B) in placental biopsies from SARS-CoV-2 negative (blue) or SARS-CoV-2 positive (orange) pregnancies. Data are presented as mean ± SEM. Differences across groups were assessed by two-way ANOVA.

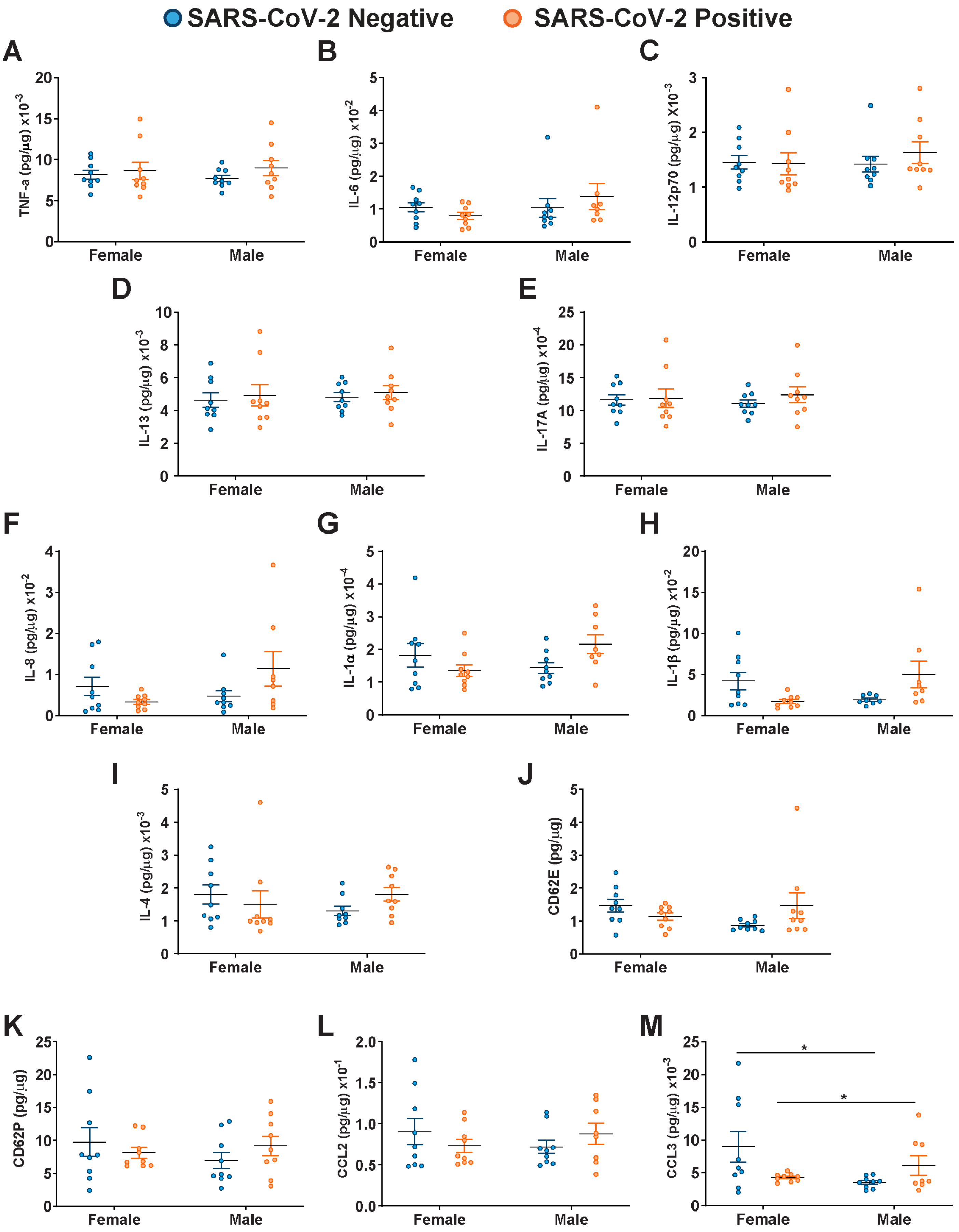
SARS-CoV-2 Negative
SARS-CoV-2 Positive
A
TNF-a (pg/µg) x10-3
B
IL-6 (pg/µg) x10-2
C
IL-12p70 (pg/µg) X10-3
D
IL-13 (pg/µg) x10-3
E
IL-17A (pg/µg) x10-4
F
IL-8 (pg/µg) x10-2
G
IL-1α (pg/µg) x10-4
H
IL-1β (pg/µg) x10-2
I
IL-4 (pg/µg) x10-3
J
CD62E (pg/µg)
K
CD62P (pg/µg)
L
CCL2 (pg/µg) x10-1
M
CCL3 (pg/µg) x10-3
*
*
Female
Male

**Fig. S10. Maternal SARS-CoV-2 infection does not impact placental cytokine expression. (A to M)** Luminex ProcartaPlex Immunoassays of tumor necrosis factor (TNF)-α (A), interleukin (IL)-6 (B), IL-12p70 (C), IL-13 (D), IL-17A (E), IL-8 (F), IL-1α (G), IL-1β (H), IL-4 (I), CD62E (J), CD62P (K), CCL2 (L), and CCL3 (M) in placental biopsies from SARS-CoV-2 negative (blue) or SARS-CoV-2 positive (orange) pregnancies. Two-way ANOVA followed by post-hoc analyses were performed to determine significance. Protein expression values are corrected to input protein concentration. Data are presented as mean ± SEM. Differences across groups were assessed by two-way ANOVA followed by Bonferroni's post-hoc analyses. $^{*}p<0.05$.

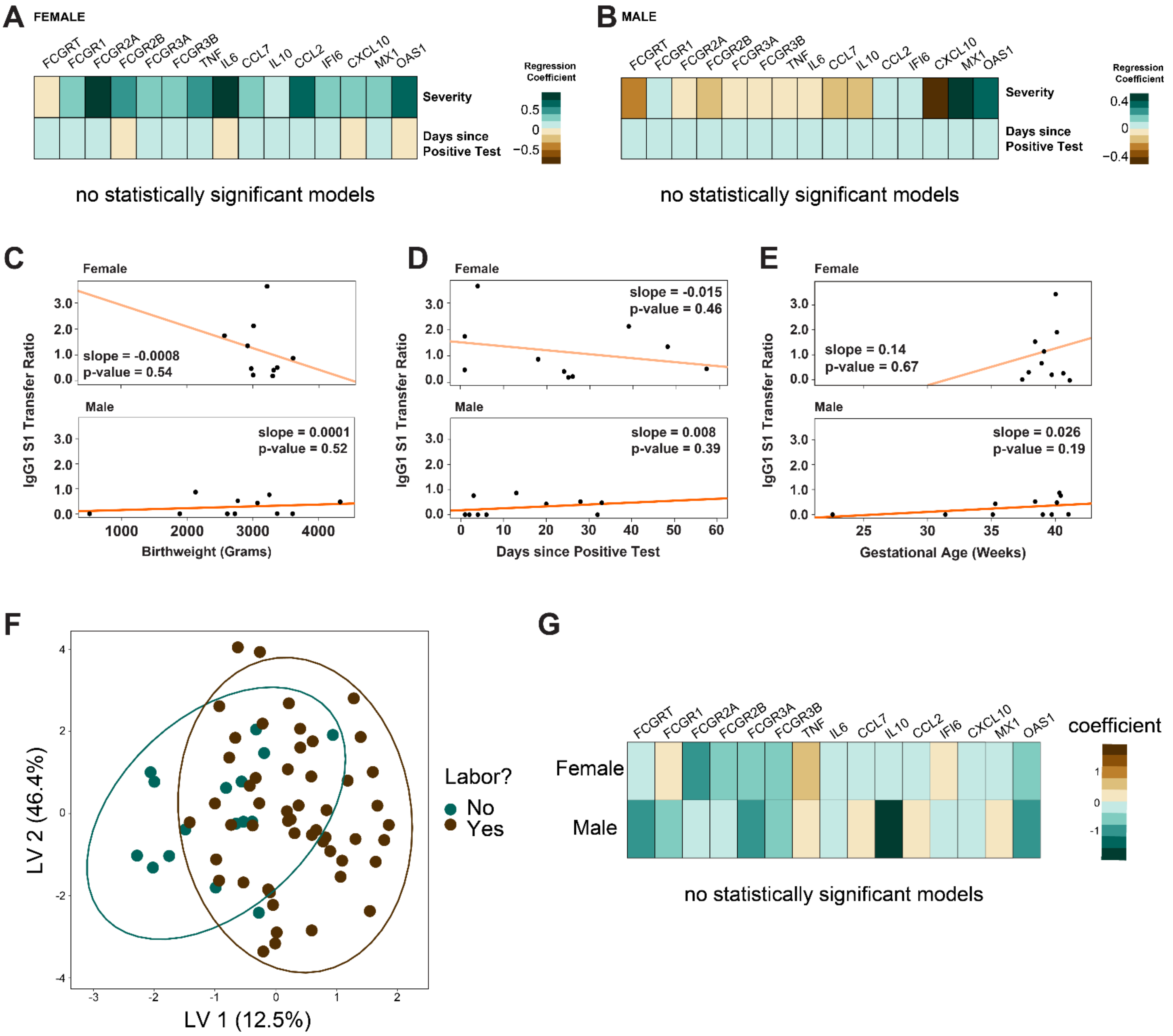


**Fig. S11. Effect of disease severity, time since infection, neonatal birthweight, gestational age, and labor status on gene expression and antibody transfer.**

**(A)** Heatmap depicting pairwise linear regressions between maternal disease severity (top), or time since positive test (bottom), and expression of each individual interferon stimulated gene (ISG) or FcR for female placentas. None of the regression models had statistical significance after false discovery rate (FDR) multiple comparisons correction using a corrected p-value of 0.05 as the cutoff. **(B)** Heatmap depicting pairwise linear regressions between maternal disease severity (top), or time since positive test (bottom), and expression of each individual ISG or FcR for male placentas. None of the regression models had statistical significance after FDR multiple comparisons correction using a corrected p-value of 0.05 as the cutoff. **(C)** Linear regression models were performed on female (top) and male (bottom) neonates separately to examine the effect of birthweight on placental transfer of S1-specific IgG1 (no significant effect). **(D)** Linear regression models were performed on pregnancies with a female fetus (top) and male fetus (bottom) separately to examine the effect of days since maternal infection on transplacental transfer of S1-specific IgG1 (no significant effect). **(E)** Linear regression models were performed on female (top) and male (bottom) neonates separately to examine the effect of

gestational age at delivery on placental transfer of S1-specific IgG1 (no significant effect). **(F)** PLSDA model demonstrating that multivariate gene expression profiles of participants to not separate by labor status. **(G)** Heatmap depicting linear regressions of fetus sex and expression of individual ISG or FcR in placentas. None of the regression models had statistical significance after FDR multiple comparisons correction using a corrected p-value of 0.05 as the cutoff.

## Supplementary Tables

**Table S1. Demographic and clinical characteristics of participants providing maternal and cord blood by fetal sex and maternal SARS-CoV-2 status.** DM/GDM, diabetes mellitus/gestational diabetes mellitus; BMI, body mass index; GA, gestational age; N/A, not applicable. [a]SARS-CoV-2 positive and negative status determined by nasopharyngeal RT PCR at time of sample collection. If a participant was SARS-CoV-2 positive at any time in pregnancy she was included in "SARS-CoV-2 positive" category. [b] Differences between groups were determined using chi-square test for categorical variables, and Kruskal-Wallis test for continuous variables presented as median [interquartile range, IQR]. [c] Disease severity classification based on published criteria from the National Institutes of Health.

| | **All (55)** | **Female** | | **Male** | | ***P***[b] |
|---|---|---|---|---|---|---|
| | | **SARS-CoV-2 negative (11)** | **SARS-CoV-2 positive (11)**[a] | **SARS-CoV-2 negative (22)** | **SARS-CoV-2 positive (11)**[a] | |
| Maternal Age, *years* | 33 [29-38] | 34 [32-38] | 37 [28-40] | 32 [29-37] | 31 [25-36] | 0.43 |
| Parity, *n* | 1 [0-2] | 1 [0-2] | 1 [0-1] | 1 [0-2] | 1 [0-2] | 0.95 |
| Race, *n (%)* | | | | | | 0.03 |
| *White* | 36 (65) | 8 (73) | 5 (45) | 18 (82) | 5 (45) | |
| *Black* | 4 (7) | 0 (0) | 3 (27) | 0 (0) | 1 (9) | |
| *Asian* | 2 (4) | 0 (0) | 0 (0) | 2 (9) | 0 (0) | |
| *Other* | 8 (15) | 3 (27) | 2 (18) | 1 (5) | 2 (18) | |
| *Not Reported* | 5 (9) | 0 (0) | 1 (9) | 1 (5) | 3 (27) | |
| Ethnicity, *n (%)* | | | | | | 0.01 |
| *Hispanic* | 19 (35) | 3 (27) | 4 (36) | 3 (14) | 9 (82) | |
| *Non-Hispanic* | 33 (60) | 7 (64) | 6 (55) | 18 (82) | 2 (18) | |
| *Not Reported* | 3 (5) | 1 (9) | 1 (9) | 1 (5) | 0 (0) | |
| Chronic hypertension, *n (%)* | 1 (2) | 0 (0) | 0 (0) | 1 (5) | 0 (0) | 0.68 |
| DM/GDM, *n (%)* | 6 (11) | 1 (9) | 1 (9) | 3 (14) | 1 (9) | 0.96 |
| BMI ≥ 30 kg/m$^2$, *n (%)* | 17 (31) | 2 (18) | 6 (55) | 5 (23) | 4 (36) | 0.21 |
| Pre-pregnancy BMI, *kg/m$^2$* | 27.0 [21.6-32.0] | 27.0 [22.6-29.8] | 29.2 [25.9-36.3] | 22.1 [20.3-30.3] | 29.5 [22.5-32.1] | 0.16 |
| GA at delivery, *weeks* | 39 [38-40] | 39 [39-39] | 40 [38-41] | 39 [38-41] | 39 [35-40] | 0.86 |
| Any labor, *n (%)* | 40 (73) | 4 (36) | 10 (91) | 16 (73) | 10 (91) | 0.01 |
| Neonatal birthweight, *g* | 3300 [3005-3630] | 3295 [3060-3590] | 3010 [2920-3315] | 3533 [3254-3784] | 3075 [2615-3610] | 0.07 |
| Multiple gestation, n | 1 | 0 | 0 | 1 | 0 | N/A |
| GA at positive SARS-CoV-2 test, *weeks* | 36.3 [32.4-38.8] | N/A | 36.3 [32.4] | N/A | 35.4 [32.4-39.6] | 0.81 |
| COVID-19 disease severity at diagnosis[c], *n* | | | | | | 0.77 |
| *Asymptomatic* | 5 (23) | N/A | 2 (18) | N/A | 3 (27) | |
| *Mild/Moderate* | 14 (64) | N/A | 7 (64) | N/A | 7 (64) | |
| *Severe/Critical* | 3 (14) | N/A | 2 (18) | N/A | 1 (9) | |
| Time between SARS-CoV-2 symptom onset and delivery, *days* | 29 [9-44] | N/A | 33 [26-58] | N/A | 29 [9-44] | 0.08 |

**Table S2. Timing of influenza and pertussis vaccination relative to maternal titers drawn at delivery hospitalization.**

| | All (55) | Female | | Male | | *P** |
|---|---|---|---|---|---|---|
| | | SARS-CoV-2 negative (11) | SARS-CoV-2 positive (11) | SARS-CoV-2 negative (22) | SARS-CoV-2 positive (11) | |
| Number of women who received Tdap vaccine, *N* | | 11 | 11 | 20 | 11 | |
| Time from Tdap (pertussis) vaccination to delivery, *days* (median, [IQR]) | 70 [58-83] | 62 [27-81] | 66 [43-84] | 75 [65-82] | 70 [48-93] | 0.50 |
| Number of women who received influenza vaccine, *N* | | 10 | 11 | 20 | 10 | |
| Time from influenza vaccination to delivery, *days* (median, [IQR]) | 200 [184-211] | 206 [196-213] | 204 [172-207] | 197 [186-213] | 191 [179-217] | 0.64 |

*Differences between groups analyzed by Kruskal-Wallis test. Continuous variables presented as median [IQR]

**Table S3. Two-way ANOVA analysis of Fc receptor gene expression, immunoblots, and immunohistochemistry.** Two-way ANOVA followed by Bonferroni's post-hoc analyses (when interaction term was significant) were performed to determine significance. All main and interaction effects for genes and proteins are represented for both male and female placentas, in addition to all post-hoc analyses performed. Significant effects are indicated by bolded red statistics. N/A: not applicable, indicates post-hoc testing not performed due to lack of significant interaction term.

| Gene | Maternal SARS-CoV-2 Status | Fetal Sex | Interaction | Bonferroni Female Neg:Pos | Bonferroni Male Neg:Pos | Bonferroni Neg Female:Male | Bonferroni Pos Female:Male |
|---|---|---|---|---|---|---|---|
| *FCGRT* | $F_{(1,63)}$ = 0.052, p = 0.82 | $F_{(1,63)}$ = 0.0037, p = 0.95 | **$F_{(1,63)}$ = 6.91, p = 0.011** | p = 0.19 | p = 0.09 | p = 0.18 | p = 0.098 |
| *FCGR1* | $F_{(1,64)}$ = 2.01, p = 0.16 | $F_{(1,64)}$ = 0.33, p = 0.57 | **$F_{(1,64)}$ = 5.64, p = 0.021** | p > 0.99 | **p = 0.019** | p = 0.46 | p = 0.060 |
| *FCGR2A* | $F_{(1,64)}$ = 1.33, p = 0.25 | $F_{(1,64)}$ = 0.017, p = 0.90 | $F_{(1,64)}$ = 1.21, p = 0.28 | n/a | n/a | n/a | n/a |
| *FCGR2B* | $F_{(1,64)}$ = 0.012, p = 0.91 | $F_{(1,64)}$ = 0.69, p = 0.41 | $F_{(1,64)}$ = 1.15, p = 0.29 | n/a | n/a | n/a | n/a |
| *FCGR3A* | $F_{(1,64)}$ = 0.43, p = 0.51 | $F_{(1,64)}$ = 0.24, p = 0.63 | **$F_{(1,64)}$ = 5.24, p = 0.025** | p = 0.51 | p = 0.042 | p = 0.14 | p = 0.36 |
| *FCGR3B* | $F_{(1,63)}$ = 3.69, p = 0.059 | $F_{(1,63)}$ = 0.031, p = 0.86 | **$F_{(1,63)}$ = 4.81, p = 0.032** | p > 0.99 | **p = 0.009** | p = 0.36 | p = 0.16 |

| Protein | Maternal SARS-CoV-2 Status | Fetal Sex | Interaction | Bonferroni Female Neg:Pos | Bonferroni Male Neg:Pos | Bonferroni Neg Female:Male | Bonferroni Pos Female:Male |
|---|---|---|---|---|---|---|---|
| FcRn | $F_{(1,78)}$ = 0.74, p = 0.39 | $F_{(1,78)}$ = 2.006, p = 0.16 | **$F_{(1,78)}$ = 5.51, p = 0.021** | p = 0.61 | **p = 0.046** | p > 0.99 | **p = 0.022** |
| FCγRI | $F_{(1,76)}$ = 0.28, p = 0.60 | $F_{(1,76)}$ = 0.67, p = 0.42 | $F_{(1,76)}$ = 1.76, p = 0.19 | n/a | n/a | n/a | n/a |
| FCγRII | $F_{(1,67)}$ = 3.79, p = 0.056 | $F_{(1,67)}$ = 0.15, p = 0.70 | $F_{(1,67)}$ = 0.41, p = 0.52 | n/a | n/a | n/a | n/a |
| FCγRIII | $F_{(1,74)}$ = 3.035, p = 0.086 | $F_{(1,74)}$ = 2.22, p = 0.14 | **$F_{(1,74)}$ = 4.86, p = 0.031** | p > 0.99 | **p = 0.011** | p > 0.99 | **p = 0.028** |

| Co-localization | Maternal SARS-CoV-2 Status | Fetal Sex | Interaction | Bonferroni Female Neg:Pos | Bonferroni Male Neg:Pos | Bonferroni Neg Female:Male | Bonferroni Pos Female:Male |
|---|---|---|---|---|---|---|---|
| FCγRIII/FcRn | $F_{(1,17)}$ = 2.27, p = 0.15 | $F_{(1,17)}$ = 0.67, p = 0.42 | **$F_{(1,17)}$ = 14.01, p = 0.002** | p = 0.24 | **p = 0.004** | **p = 0.016** | p = 0.078 |
| FCγRII/FcRn | $F_{(1,19)}$ = 0.86, p = 0.37 | $F_{(1,19)}$ = 0.034, p = 0.86 | $F_{(1,19)}$ = 1.15, p = 0.30 | n/a | n/a | n/a | n/a |

| | | | | | | | |
|---|---|---|---|---|---|---|---|
| FCγRI/FcRn | $F_{(1,19)}$ = 0.0004, p = 0.99 | $F_{(1,19)}$ = 0.59, p = 0.45 | $F_{(1,19)}$ = 0.017, p = 0.90 | n/a | n/a | n/a | n/a |

**Table S4. Two-way ANOVA analysis of inflammatory cytokine and interferon stimulated gene expression and CD163 immunohistochemistry.** Two-way ANOVA followed by Bonferroni's post-hoc analyses were performed to determine significance. All main and interaction effects for genes of interest are represented for both fetal males and fetal females in addition to all post-hoc analyses performed. Significant effects are indicated in bolded red. n/a, not applicable.

| Gene | Maternal Covid Status | Fetal Sex | Interaction | Bonferroni Female Neg:Pos | Bonferroni Male Neg:Pos | Bonferroni Neg Female:Male | Bonferroni Pos Female:Male |
|---|---|---|---|---|---|---|---|
| *TNF* | $F_{(1,63)}$ = 0.061, p =0.81 | $F_{(1,63)}$ = 0.31 , p = 0.58 | $F_{(1,63)}$ = 0.076, p = 0.78 | n/a | n/a | n/a | n/a |
| *IL6* | $F_{(1,64)}$ = 0.66, p = 0.42 | $F_{(1,64)}$ = 0.017, p = 0.90 | $F_{(1,64)}$ = 2.3, p = 0.13 | n/a | n/a | n/a | n/a |
| *CCL7* | $F_{(1,63)}$ = 0.0075, p = 0.93 | $F_{(1,63)}$ = 0.57 , p = 0.45 | $F_{(1,63)}$ = 1.71 , p = 0.20 | n/a | n/a | n/a | n/a |
| *IL10* | $F_{(1,63)}$ = 3.58, p = 0.063 | $F_{(1,63)}$ = 0.19, p = 0.66 | **$F_{(1,63)}$ = 7.72, p = 0.0072** | p > 0.9999 | **p = 0.0030** | p = 0.068 | p = 0.17 |
| *CCL2* | **$F_{(1,64)}$ = 4.69, p = 0.034** | $F_{(1,64)}$ = 0.85, p = 0.36 | $F_{(1,64)}$ = 1.25, p = 0.27 | p = 0.93 | **p = 0.047** | p > 0.99 | p = 0.26 |
| *IFI6* | $F_{(1,64)}$ = 0.25, p = 0.62 | $F_{(1,64)}$ = 0.17, p = 0.68 | **$F_{(1,64)}$ = 7.74, p = 0.0071** | p = 0.22 | **p = 0.047** | p = 0.24 | **p = 0.038** |
| *CXCL10* | $F_{(1,62)}$ = 3.049, p = 0.086 | $F_{(1,62)}$ = 0.17, p = 0.68 | **$F_{(1,62)}$ = 12.36, p = 0.0008** | p = 0.42 | **p = 0.0010** | **p = 0.022** | **p = 0.045** |
| *MX1* | **$F_{(1,63)}$ = 4.46, p = 0.039** | $F_{(1,63)}$ = 0.12, p = 0.74 | $F_{(1,63)}$ = 1.038, p = 0.31 | p = 0.89 | p = 0.059 | p > 0.99 | p = 0.63 |
| *OAS1* | $F_{(1,63)}$ = 0.74, p = 0.39 | $F_{(1,63)}$ = 0.039, p = 0.85 | **$F_{(1,63)}$ = 5.99, p = 0.017** | p = 0.54 | **p = 0.044** | p = 0.27 | p = 0.11 |
| *YWHAZ* | $F_{(1,63)}$ = 0.00017, p = 0.99 | $F_{(1,63)}$ = 0.0018, p = 0.97 | $F_{(1,63)}$ = 0.54, p = 0.46 | n/a | n/a | n/a | n/a |
| *TOP1* | $F_{(1,44)}$ = 0.53, p = 0.47 | $F_{(1,44)}$ = 0.0026, p = 0.96 | $F_{(1,44)}$ = 0.0098, p = 0.92 | n/a | n/a | n/a | n/a |
| | | | | | | | |
| **Protein** | **Maternal Covid Status** | **Fetal Sex** | **Interaction** | **Bonferroni Female Neg:Pos** | **Bonferroni Male Neg:Pos** | **Bonferroni Neg Female:Male** | **Bonferroni Pos Female:Male** |
| IFNα | $F_{(1,32)}$ = 1.88, p = 0.18 | $F_{(1,32)}$ = 0.42, p = 0.52 | **$F_{(1,32)}$ = 4.23, p = 0.048** | p > 0.99 | **p = 0.042** | p = 0.65 | p = 0.13 |
| IFNγ | $F_{(1,32)}$ = 2.14, p = 0.15 | $F_{(1,32)}$ = 1.94, p = 0.17 | **$F_{(1,32)}$ = 7.00, p = 0.013** | p = 0.81 | **p = 0.013** | p = 0.77 | **p = 0.015** |

| | | | | | | | |
|---|---|---|---|---|---|---|---|
| CXCL10 | $F_{(1,32)} = 0.043, p = 0.84$ | $F_{(1,32)} = 0.084, p = 0.77$ | **$F_{(1,32)} = 12.82, p = 0.0011$** | **$p = 0.023$** | **$p = 0.046$** | **$p = 0.043$** | **$p = 0.020$** |
| CCL4 | $F_{(1,32)} = 0.14, p = 0.71$ | $F_{(1,32)} = 0.27, p = 0.61$ | **$F_{(1,32)} = 12.09, p = 0.0015$** | **$p = 0.020$** | **$p = 0.039$** | **$p = 0.016$** | **$p = 0.048$** |
| TNFα | $F_{(1,32)} = 1.27, p = 0.27$ | $F_{(1,32)} = 0.0057, p = 0.94$ | $F_{(1,32)} = 0.26, p = 0.62$ | n/a | n/a | n/a | n/a |
| IL-6 | $F_{(1,32)} = 0.029, p = 0.87$ | $F_{(1,32)} = 1.29, p = 0.26$ | $F_{(1,32)} = 1.47, p = 0.24$ | n/a | n/a | n/a | n/a |
| IL-12p70 | $F_{(1,32)} = 0.29, p = 0.59$ | $F_{(1,32)} = 0.25, p = 0.62$ | $F_{(1,32)} = 0.49, p = 0.49$ | n/a | n/a | n/a | n/a |
| IL-13 | $F_{(1,32)} = 0.38, p = 0.54$ | $F_{(1,32)} = 0.15, p = 0.71$ | $F_{(1,32)} = 0.00021, p = 0.99$ | n/a | n/a | n/a | n/a |
| IL-17A | $F_{(1,32)} = 0.58, p = 0.45$ | $F_{(1,32)} = 0.00046, p = 0.98$ | $F_{(1,32)} = 0.30, p = 0.59$ | n/a | n/a | n/a | n/a |
| IL-8 | $F_{(1,32)} = 0.40, p = 0.53$ | $F_{(1,32)} = 1.44, p = 0.24$ | **$F_{(1,32)} = 4.85, p = 0.035$** | $p > 0.99$ | $p = 0.34$ | $p > 0.99$ | $p = 0.15$ |
| IL-1α | $F_{(1,31)} = 0.25, p = 0.63$ | $F_{(1,31)} = 0.66, p = 0.42$ | **$F_{(1,31)} = 5.31, p = 0.028$** | $p > 0.99$ | $p = 0.36$ | $p > 0.99$ | $p = 0.23$ |
| IL-1β | $F_{(1,30)} = 0.095, p = 0.76$ | $F_{(1,30)} = 0.29, p = 0.59$ | **$F_{(1,30)} = 8.32., p = 0.0072$** | $p = 0.42$ | $p = 0.22$ | $p = 0.64$ | $p > 0.99$ |
| IL-4 | $F_{(1,32)} = 0.14, p = 0.71$ | $F_{(1,32)} = 0.11, p = 0.74$ | $F_{(1,32)} = 2.060, p = 0.16$ | n/a | n/a | n/a | n/a |
| CD62E | $F_{(1,32)} = 0.34, p = 0.57$ | $F_{(1,32)} = 0.33, p = 0.57$ | $F_{(1,32)} = 3.49, p = 0.071$ | n/a | n/a | n/a | n/a |
| CD62P | $F_{(1,32)} = 0.043, p = 0.84$ | $F_{(1,32)} = 0.34, p = 0.56$ | $F_{(1,32)} = 1.62, p = 0.21$ | n/a | n/a | n/a | n/a |
| CCL2 | $F_{(1,31)} = 0.0028, p = 0.96$ | $F_{(1,31)} = 0.029, p = 0.87$ | $F_{(1,31)} = 2.046, p = 0.16$ | n/a | n/a | n/a | n/a |
| CCL3 | $F_{(1,31)} = 0.57, p = 0.46$ | $F_{(1,31)} = 1.66, p = 0.21$ | **$F_{(1,31)} = 6.85, p = 0.014$** | **$p = 0.043$** | $p = 0.41$ | **$p = 0.017$** | $p = 0.72$ |
| | | | | | | | |
| **Gene** | **Maternal Covid Status** | **Fetal Sex** | **Interaction** | **Bonferroni Female Neg:Pos** | **Bonferroni Male Neg:Pos** | **Bonferroni Neg Female:Male** | **Bonferroni Pos Female:Male** |
| %CD163 + cells | **$F_{(1,22)} = 4.36 , p = 0.049$** | $F_{(1,22)} = 1.67, p = 0.21$ | $F_{(1,22)} = 2.59 , p = 0.12$ | $p > 0.99$ | **$p = 0.025$** | $p = 0.97$ | $p = 0.071$ |

**Table S5. Taqman gene expression assays used for RT-qPCR.** Thermo Fisher Scientific Assay ID, Gene symbol, and color dye for each gene expression assay used are shown.

| Assay ID | Gene Symbol | Dye Label |
|---|---|---|
| Hs00237047_m1 | *YWHAZ* | VIC_PL |
| Hs00175415_m1 | *FCGRT* | FAM-MGB |
| Hs00174081_m1 | *FCGR1A* | FAM-MGB |
| Hs00234969_m1 | *FCGR2A* | FAM-MGB |
| Hs00269610_m1 | *FCGR2B* | FAM-MGB |
| Hs02388314_m1 | *FCGR3A* | FAM-MGB |
| Hs04334165_m1 | *FCGR3B* | FAM-MGB |
| Hs00234140_m1 | *CCL2* | FAM-MGB |
| Hs00174131_m1 | *IL6* | FAM-MGB |
| Hs00174128_m1 | *TNF* | FAM-MGB |
| Hs00171147_m1 | *CCL7* | FAM-MGB |
| Hs00242571_m1 | *IFI6* | FAM-MGB |
| Hs00171042_m1 | *CXCL10* | FAM-MGB |
| Hs00895608_m1 | *MX1* | FAM-MGB |
| Hs00961622_m1 | *IL10* | FAM-MGB |
| Hs00242943_m1 | *OAS1* | FAM-MGB |
| Hs00243257_m1 | *TOP1* | VIC_PL |
